# Ligand Substitution in Three Organic–Inorganic Hybrid Zincophosphite Analogs: Modulating Lanthanide Affinity and White-Light Emission via Hard–Soft Acid–Base Interplay

**DOI:** 10.1021/acs.inorgchem.6c04380

**Published:** 2026-09-12

**Authors:** Mu-Chien Yin, Chih-Feng Yen, Tzu-Yuan Sung, Ju-Ying Chen, Jui Hsieh, Chun-Ru Hsu, Wei-Cheng Chen, Ming-Der Wu, Chih-Min Wang

**Affiliations:** † Department of Bioscience and Biotechnology, 34880 National Taiwan Ocean University, Keelung 202301, Taiwan, R.O.C; ‡ Department of Microelectronics Engineering, 554423 National Kaohsiung University of Science and Technology, Kaohsiung 811213, Taiwan, R.O.C; § Department of Graduate Institute of Aquaculture, National Kaohsiung University of Science and Technology, Kaohsiung 811213, Taiwan, R.O.C; ∥ Bioresource Collection and Research Center, 63418 Food Industry Research and Development Institute, Hsinchu 300197, Taiwan, R.O.C

## Abstract

Systematically modulating the structural environment of solid-state materials is critical for tailoring physical–chemical interactions. Here, we demonstrated the synthesis and characteristics of three hybrid zincophosphite frameworks (**NTOU-12a**, **-12b**, and **-12c**) that were designed by using Hard–Soft (HSAB) principles to modulate lanthanide adsorption and luminescence. In the replacement of oxygen-donor dicarboxylate ligands with a sulfur-containing linker (2,5-thiophenedicarboxylic acid), the local coordination environment and structural chemistry were systematically modified while preserving the overall framework architecture. This substitution led to 27–42% and 32–41% decreases in uptake of Eu^3+^ and Tb^3+^ ions, respectively. By tuning the ratio of adsorbed Eu^3+^/Tb^3+^ on the title compounds, the emission color could be continuously tuned over a wide spectral range. Notably, **NTOU-12a** exhibited white-light emission under 254 nm excitation. These results demonstrate that rational ligand substitution provides an effective strategy for designing crystalline materials with selective lanthanide adsorption and tunable multicolor luminescence.

## Graphical abstract

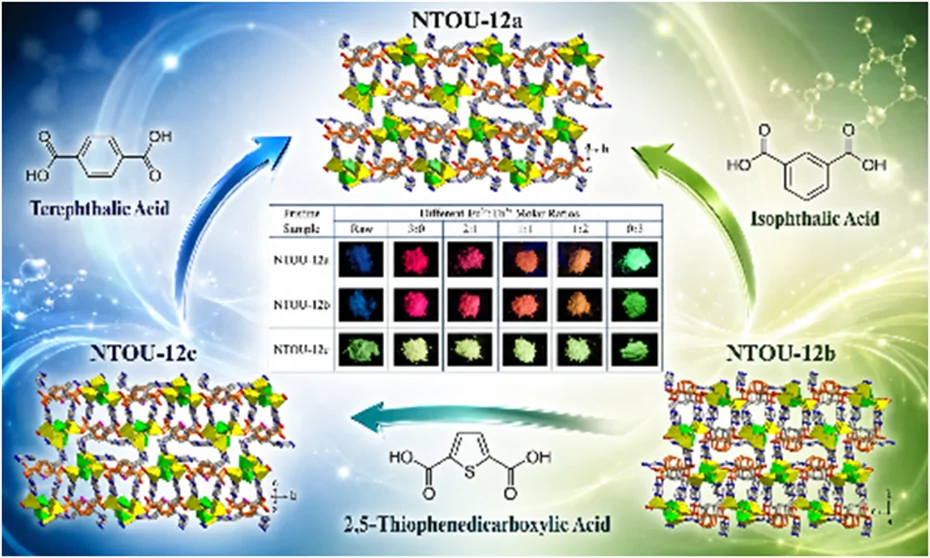

## Introduction

Lanthanides (Lns) have become a critical resource for the needs of modern technology, such as clean energy, luminescence, and defense industries etc.^1–4^ Accordingly, crystalline lanthanide-based compounds are extensively investigated for chemical sensing, adsorption, separation, and tunable photoluminescence.^5–7^ For example, Chen’s team established a prototypical luminescent metal-organic framework (MOF) where precisely engineered pore microenvironments dictate specific host-guest interactions, enabling the highly selective sensing of anions;^8^ the mixed-metal-mixed-ligand LnMOFs functioned as ratiometric luminescence thermometers in a wide temperature range of cryogenic 10–110 K and ambient 70–330 K temperatures;^9^ Zhou’s group reported a Eu-MOF containing 2,5-furandicarboxylic acid served as the foundational platform and specifically accommodated different analytes with particular structures and properties by the replacement of distinct hexacyclic isophthalic acids as the modules.^10^ In those cases, confining different kinds of and specific ratios of lanthanide ions within solid-state materials of coordination polymers (CPs), MOFs, metal phosphites or/and phosphates relies on direct synthesis or/and post-synthetic modification. As a result, the microenvironments tailor the chemical/physical properties, giving fascinating applications. For now, much research is devoted to the design, synthesis, and application of lanthanide-based materials, as well as recycling and purifying lanthanides from waste. The wide applications and limited sources (mostly from China, United States, and a few other countries) show the real importance of this research. This situation also brings up issues of supply chain vulnerability and lanthanides as critical strategic resources.^11^ To reduce environmental pollution and energy consumption, adsorption is considered an effective method for Ln recovery.^12–14^ Recently, Bu’s group synthesized a series of metal-organic frameworks (MOFs) that exhibited excellent selectivity for different Ln ions, providing a cost-effective method for Ln separation.^15^ The rational design of functional coordination materials relies heavily on the selection for appropriate organic linkers and their matchings. Dicarboxylic acid–based ligands are widely used in framework construction due to their versatile coordination modes and structural rigidity. Variations in the geometry, length, and functional groups of dicarboxylate linkers affect the framework topology and structural diversity. Moreover, tailoring the structural backbone with specific dicarboxylic acids and functional groups enables the modulation of physicochemical properties, providing a platform for diverse applications ranging from selective adsorption to advanced electrochemical and luminescent devices.^16–19^ Motivated by practical applications and structural design principles, we synthesized three organic-inorganic hybrid mixed-ligand zincophosphites. The deliberate incorporation of inorganic phosphite anions and a complex nitrogen-donor ligand (TIMB) alongside dicarboxylates is central to our design strategy. Although the simultaneous assembly of those components significantly increases the difficulty of crystal growth, it uniquely elevates the structural complexity and the probability of discovering novel topologies. The phosphite groups act as robust inorganic nodes capable of bridging multiple metal centers to form stable inorganic chains, while the highly flexible, multidentate TIMB ligand serves as an essential cross-linker to expand the structures with more complex motifs and/or frameworks. The different coordination environments and surface features within these frameworks dictate their distinct lanthanide adsorption behaviors and tunable luminescent properties. Initially, we isolated two highly stable crystals, **NTOU-12a** and **NTOU-12b**, incorporating terephthalic acid and isophthalic acid, respectively; both exhibit blue emission under 365 nm UV irradiation. Subsequently, we synthesized **NTOU-12c** by substituting the initial ligands with 2,5-thiophenedicarboxylic acid. This strategic modification was guided by the Hard and Soft Acids and Bases (HSAB) theory.^20^ While Yaghi’s pioneering multivariate MOF concept integrates diverse functional groups into a single continuous lattice,^21^ our work relies on systematic linker substitution to generate a family of structurally analogous frameworks (**NTOU-12a**, **-12b**, and **-12c**) to investigate the role of ligand replacement in modulating material performance. These design principles establish a robust foundation for engineering stable crystalline materials with tailored adsorption and luminescent profiles. While **NTOU-12a** and **NTOU-12b** exhibit highly tunable luminescence spanning the visible spectrum (including white light) under 254 nm excitation, **NTOU-12c** displays a distinct green emission. By strategically replacing traditional oxygen- or nitrogen-donor ligands with sulfur-containing analogs, we observed that **NTOU-12c** exhibited a 27–42% reduction in the adsorption of Eu^3+^ and a 32–41% reduction for Tb^3+^. The lower Ln^3+^ uptake observed for NTOU-12c is consistent with an HSAB-based interpretation, while structural and surface-related factors may also contribute to the adsorption behavior. Herein, the development of these diverse hybrid zincophosphites highlights the dual significance of structural innovation and functional integration. By successfully coupling tailored adsorption capacities with tunable luminescence, these strategically engineered frameworks provide a versatile platform, paving the way for adsorption of critical lanthanide resources from complex environments, as well as the development of new multifunctional optical devices and chemical sensors.

## Experimental Section

### Synthesis

Three colorless crystalline compounds were synthesized using similar hydro­(solvo)­thermal procedures. The reactions were carried out in 23 mL Teflon-lined stainless-steel Parr autoclaves heated at 150 °C for 2 days. For synthesis of **NTOU-12a**, a general mixture contains Zn­(NO_3_)_2_·6H_2_O (0.25 mmol, 75.9 mg), H_3_PO_3_ (0.50 mmol, 41.8 mg), 48% aqueous HF (3.0 mmol, 109 μL), and 1,2,4,5-tetrakis­((1*H*-imidazol-1-yl)­methyl)­benzene (TIMB, 0.25 mmol, 101.7 mg), terephthalic acid (TPA, 0.20 mmol, 33.6 mg), H_2_O (5.0 mL) and DMF (2.0 mL). For **NTOU-12b**, the synthesis protocol was similar to that of **NTOU-12a** with the mixture of Zn­(NO_3_)_2_·6H_2_O (0.25 mmol, 75.9 mg), H_3_PO_3_ (0.50 mmol, 41.8 mg), 48% aqueous HF (3.0 mmol, 109 μL), and 1,2,4,5-tetrakis­((1*H*-imidazol-1-yl)­methyl)­benzene (TIMB, 0.25 mmol, 101.7 mg), isophthalic acid (IPA, 0.20 mmol, 33.6 mg) H_2_O (5.5 mL) and DMF (1.5 mL). For **NTOU-12c**, the procedure was performed by heating the mixture of Zn­(NO_3_)_2_·6H_2_O (0.25 mmol, 75.9 mg), H_3_PO_3_ (0.50 mmol, 41.8 mg), 48% aqueous HF (3.0 mmol, 109 μL), and 1,2,4,5-tetrakis­((1*H*-imidazol-1-yl)­methyl)­benzene (TIMB, 0.20 mmol, 81.3 mg), 2,5-thiophenedicarboxylic acid (TPDC, 0.20 mmol, 34.8 mg) H_2_O (6.0 mL) and DMF (1.0 mL). Minor adjustments to the solvent volumes were made to accommodate the varying solubilities of organic carboxylate ligands. Those crystals were filtered, washed with water and ethanol, and dried in an oven.

### Lanthanide Adsorption Experiments

For the luminescence tuning experiments, finely ground powder samples (0.03 mmol) of the synthesized compounds (27.6, 29.2, and 27.8 mg for **NTOU-12a**, **-12b**, and **-12c**, respectively) were stirred in 5.0 mL of a 20 mM lanthanide water/ethanol solution at room temperature for 2 h. The molar ratios of Eu^3+^/Tb^3+^ in the solutions were systematically varied as 3/0, 2/1, 1/1, 1/2, and 0/3. Furthermore, in the effort to achieve white-light emission, the pristine powder samples (0.03 mmol) were treated with 5.0 mL of a 12 mM lanthanide water/ethanol solution specifically prepared with an Eu^3+^/Tb^3+^ molar ratio of 1/4 under identical conditions. The resulting Ln^3+^-adsorbed products were subsequently collected by filtration (or centrifugation), washed thoroughly with ethanol, and dried prior to photoluminescence and ICP-OES analyses.

### Initial Characterization

Powder X-ray diffraction (PXRD) data were collected by a Bruker D2 Phaser X-ray diffractometer, and the patterns of the bulk products were matched with the calculated ones derived from single-crystal X-ray diffraction (SCXRD) data for the three compounds (Figures S1–S3). Elemental analysis (EA) results were in good agreement with the expected chemical formulas for all three compounds. The isolated yields ranged from 77% to 85% based on Zn. Anal. Found (calcd) for **NTOU-12a**: C, 39.05% (39.13%); H, 3.05% (3.28%); N, 12.14% (12.17%); for **NTOU-12b**: C, 36.83% (36.96%); H, 3.31% (3.72%); N, 11.43% (11.50%); **NTOU-12c**: C, 36.10% (36.29%); H, 2.71% (3.05%); N, 12.11% (12.09%). Thermogravimetric analysis (TGA) of the three compounds was performed on powder samples using a PerkinElmer TGA7 thermal analyzer under a flow of oxygen, heating from 40 to 900 °C at a rate of 10 °C/min (Figures S4–S6). For **NTOU-12a**, a substantial weight loss of 57.20% occurred in several overlapping steps between 40 and 900 °C. In the case of **NTOU-12b**, an initial weight loss of 4.86% between 40 and 200 °C corresponds to the presence of three lattice water molecules per formula unit (5.54%). Total weight loss of 57.07% commenced approximately from 40 °C to 900 °C. **NTOU-12c** exhibited a major overlapping weight loss of 58.12% from 40 to 900 °C. The final decomposition products from the TG analysis were indicated as Zn_3_(PO_4_)_2_ (JCPDS: 29-1390 for **NTOU-12a** and **12b**; 76-0518 for **NTOU-12c**) by PXRD. The theoretical total weight losses for the three compounds are calculated to be 58.06%, 60.39%, and 58.34%, respectively. The discrepancy between the calculated and observed total weight losses in the 40–900 °C range is attributed to the oxidation of phosphorus to phosphate groups during decomposition (For **NTOU-12a**, 2.93% + 57.20% = 60.13%; For **NTOU-12b**, 2.78% + 57.07% = 59.85%; For **NTOU-12c**, 2.92% + 58.12% = 61.04%). According to the TGA and PXRD analyses (Figures S7–S9), three compounds of **NTOU-12a-12c** maintained the structural framework up to 400, 300, and 350°C, respectively. Furthermore, as shown in Figures S10–S12, these materials exhibited robust chemical stability in water and aqueous solutions over a broad pH range (pH 2–11 for **NTOU-12a;** pH 4–12 for **NTOU-12b**; pH 2–11 for **NTOU-12c**) (Figures S10–S12). For single-crystal X-ray crystallography, suitable colorless crystals were selected for indexing and intensity data collection on a Bruker X8 Apex2 CCD diffractometer equipped with a normal-focus, 3 kW sealed-tube X-ray source. The number (8224) of unique reflections (*F*_0_ > 2σ­(*F*_0_)) was collected with 2θ_max_ = 28.28° and *R*_int_ = 0.0731 for **NTOU-12a** (27.56° and 0.0724 for **NTOU-12b**; 27.01° and 0.0428 for **NTOU-12c**, respectively). Absorption corrections were applied using the SADABS program. Based on systematic absences, successful structure solutions, and refinements, the space group for all three compounds was determined to be *P*2_1_/*n* (No. 15). The structures were solved using a direct method and all atoms were located in general positions. The metal and phosphorus atoms were identified first, followed by the O, C, N, and S atoms, which were from difference Fourier maps. Hydrogen atoms bonded to the carbon atoms of organic molecules were positioned geometrically and refined using a riding model. Each phosphorus atom in three compounds adopts a tetrahedral geometry, bonding to three zinc atoms through oxygen atoms, leaving one P–H terminal group. On the basis of the results for SCXRD, TGA, and EA analyses, the chemical formula of **NTOU-12a**, **-12b**, and **-12c** can be determined as Zn_3_(TIMB)­(TPA)­(HPO_3_)_2_, Zn_3_(TIMB)­(IPA)­(HPO_3_)_2_·3H_2_O, Zn_3_(TIMB)­(TPDC)­(HPO_3_)_2_, respectively. Crystal data of **NTOU-12a**: monoclinic, space group *P*2_1_/*n*, *Z* = 4, Mr = 918.65 g mol^–1^, and *a* = 8.5109(5) Å, *b* = 14.649(1) Å, *c* = 26.784(2) Å, β = 97.308(3)°, *V* = 3312.3(4) Å^3^, *D*_calc_ = 1.950 g cm^–3^, λ = 1.54184 A, μ = 2.324 mm^–1^, 8224 unique reflections with [*I* > 2σ­(*I*)], *R*_1_ = 0.0271, *w*R**_2_ = 0.0538, *S* = 1.045; **NTOU-12b**: monoclinic, space group *P*2_1_/*n*, *Z* = 4, Mr = 918.65 g mol^–1^, and *a* = 9.2246(3) Å, *b* = 29.837(1) Å, *c* = 13.8204(5) Å, β = 97.164(2)°, *V* = 3774.2(2) Å^3^, *D*_calc_ = 1.617 g cm^–3^, λ = 1.54184 A, μ = 2.040 mm^–1^, 8492 unique reflections with [*I* > 2σ­(*I*)], *R*_1_ = 0.0473, *w*R**_2_ = 0.0933, *S* = 1.025; **NTOU-12c**: monoclinic, space group *P*2_1_/*n*, *Z* = 4, Mr = 924.68 g mol^–1^, and *a* = 8.4490(4) Å, *b* = 14.5463(6) Å, *c* = 27.2867(14) Å, β = 97.524(2)°, *V* = 3324.7(3) Å^3^, *D*_calc_ = 1.847 g cm^–3^, λ = 1.54184 A, μ = 2.376 mm^–1^, 7638 unique reflections with [*I* > 2σ­(*I*)], *R*_1_ = 0.0365, *w*R**_2_ = 0.075, *S* = 1.015. All calculations were performed using the Bruker SHELXTL software package.

## Results and Discussion

### Structure

Crystallographic analyses reveal analogous coordination architectures across all frameworks, each comprising three zinc cations, two phosphite groups, and discrete nitrogen- and oxygen-donor ligands. As illustrated in Figure S13, the zinc centers adopt four-coordinate tetrahedral geometries, specifically forming [ZnNO_3_] and [ZnN_2_O_2_] tetrahedra. The Zn(1) center is coordinated by two phosphite oxygen atoms and two nitrogen atoms from the TIMB linkers. Conversely, both Zn(2) and Zn(3) coordinate to two phosphite oxygen atoms, one TIMB nitrogen atom, and one oxygen atom derived from the respective carboxylate ligands (TPA, IPA, or TPDC). Furthermore, each phosphorus atom bridges three zinc cations via its oxygen atoms, with the P–H bond remaining as a terminal group. The Zn(2) and Zn(3) tetrahedra each share two oxygen atoms (O(1) and O(2); O(4) and O(5), respectively) with two P atoms to form a zincophosphite tetramer with one four-membered ring.

The structural building unit is subsequently linked to a one-dimensional inorganic chain via two oxygen atoms (O(3) and O(6)) of the Zn(1) atom extending in the opposite direction (Figure 1). The organic oxygen-donor ligands bridge the Zn atoms of the inorganic chains to form carboxylate-zincophosphite layers with varying window sizes. The ligands coordinate in a bis-monodentate fashion, adopting a *cis* conformation in **NTOU-12a** and **NTOU-12c**, and a *trans* configuration in **NTOU-12b**. The crystallographic atom-to-atom distances between adjacent chains and ligands in the windows are 14.7 Å × 8.5 Å for **NTOU-12a**, 13.8 Å × 9.2 Å for **NTOU-12b**, and 14.6 Å × 8.5 Å for **NTOU-12c** (Figure 1). In previous reports, the terephthalate (TPA) ligand exhibited five distinct coordination modes within the crystalline compounds: bis-monodentate, mono-bidentate, as well as chelating, bridging, and chelating/bridging bis-bidentate.^22–25^ Among these binding modes, the bis-monodentate configuration featuring a *cis*-coordination linkage between the metal centers and carboxyl groups is rarely observed. Interestingly, the first time this specific TPA linkage has been obtained in a mixed-ligand zincophosphite, although we previously reported the same linkage in a hybrid zinc phosphate synthesized in the presence of 1,7-diaminoheptane.^26^ It is noteworthy that while the three title compounds display similar formulas and overarching structures, their microstructural variations can be attributed to the addition of a second nitrogen-donor ligand and their distinct geometrical configurations of TPA, IPA, and TPDC. From the perspective of the carboxylate-zincophosphite layers, the three compounds exhibit distinct differences in the binding modes of the oxygen-donor ligands, the degree of distortion within the zigzag metallophosphite chains, and the sizes of the window openings (Figure 1). The coordination of the multidentate nitrogen-donor ligands to the zinc atoms facilitates the expansion of the two-dimensional layers into a three-dimensional framework. The diverse porous structures are lined with multiple types of atoms, giving rise to their distinct geometrical and functional profiles (Figure 2). Within these frameworks, the topological cross sections of the structural voids exhibit distinct crystallographic dimensions of 11.9 Å × 4.9 Å, 12.9 Å × 4.4 Å, and 11.7 Å × 4.9 Å for **NTOU-12a**, **NTOU-12b**, and **NTOU-12c**, respectively. These structural variations are primarily driven by the differing configurations and coordination modes of the dicarboxylate ligands, which dictate their internal microstructures. While **NTOU-12b** contains three lattice water molecules, both structures of **NTOU-12a** and **NTOU-12c** are denser without any solvent residues. PLATON calculations also reveal a solvent-accessible volume of 8.4% per unit cell for **NTOU-12b** and confirm the absence of any accessible voids for solvent molecules in **NTOU-12a** and **NTOU-12c**. The surfaces, defects and external microenvironments might govern the frameworks’ capacity to absorb lanthanide ions, thereby laying the foundation for their unique luminescent properties, which are discussed in the following section.

**Figure 1:**
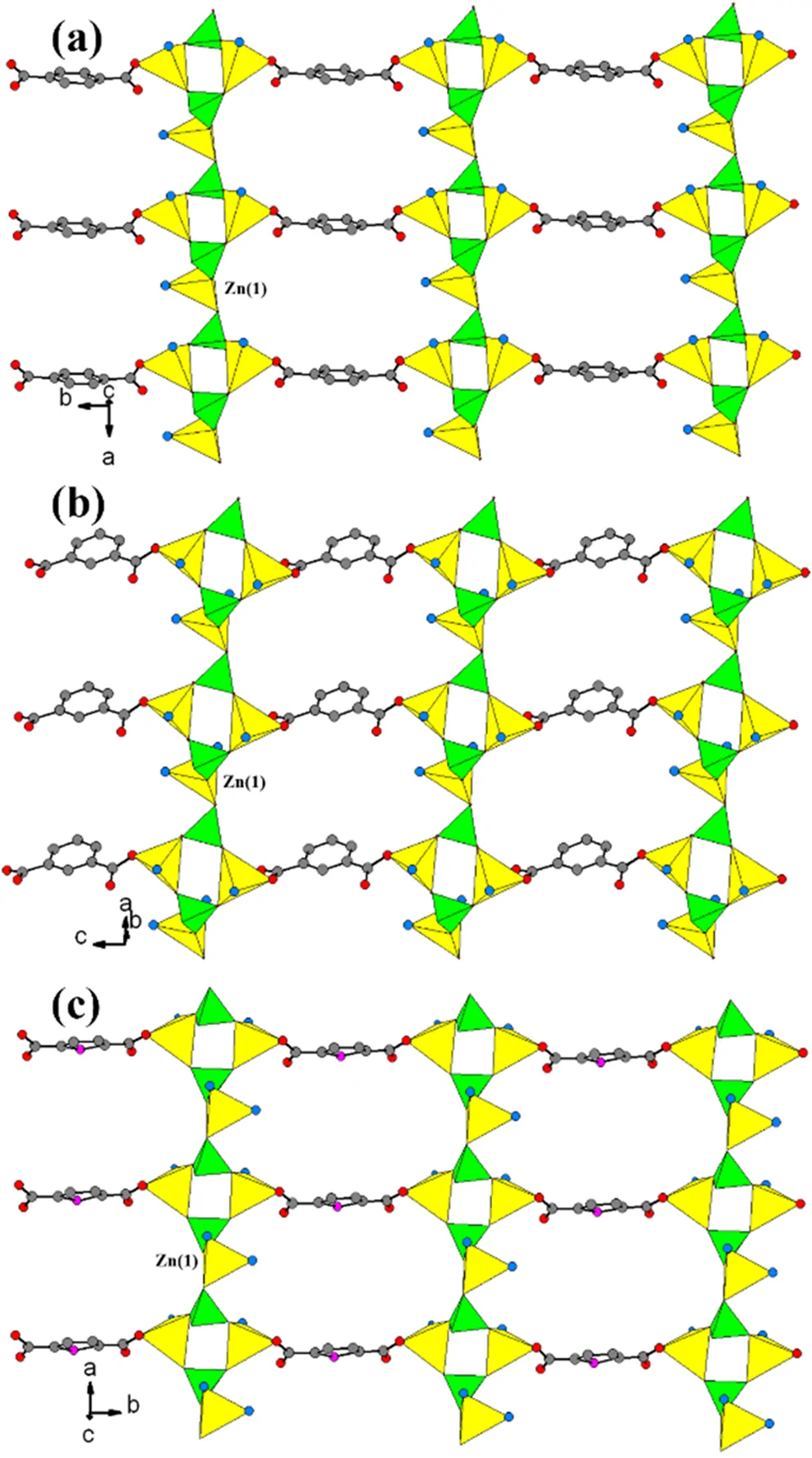
Structural components in constructing carboxylate-zincophosphite layers for **NTOU-12a** (a), **NTOU-12b** (b), and **NTOU-12c** (c). One-dimensional inorganic chains consisting of tetramers and [ZnO_2_N_4_] bridging units. The window sizes were estimated based on the adjacent atoms of the building groups. Blue, gray, and red circles represent nitrogen (N), carbon (C), and oxygen (O) atoms, respectively. Phosphorus and zinc polyhedra are depicted in green and yellow, respectively. Hydrogen atoms (H) are omitted for visual simplicity.

**Figure 2:**
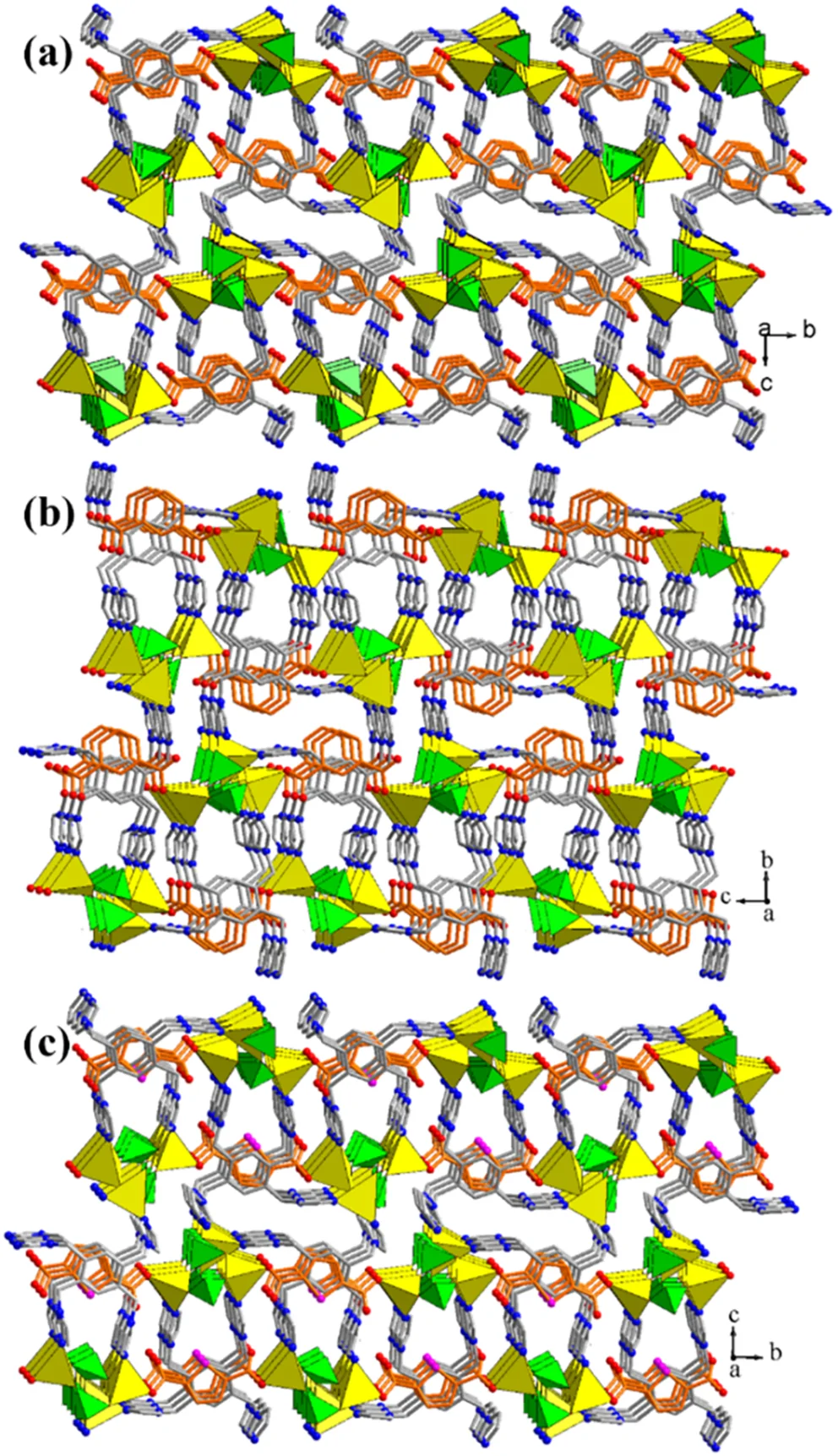
Perspective views of the structures for **NTOU-12a** (a), **NTOU-12b** (b), and **NTOU-12c** (c). The three-dimensional frameworks are constructed from carboxylate-zincophosphite layers and TIMB linkers, showing the multiple voids and structural features.

### Luminescence and Adsorption

Despite sharing similar components and structural frameworks, the three title compounds exhibited distinct photoluminescence characteristics. Under 365 nm UV irradiation, all three compounds displayed blue emission. Interestingly, upon excitation at 254 nm, **NTOU-12c** exhibited green emission, whereas **NTOU-12a** and **NTOU-12b** produced hazy-blue emissions. To gain deeper insight into the photophysical origins of these distinct luminescent behaviors, the solid-state emission spectra of the title compounds and their corresponding free ligands were thoroughly investigated at room temperature (Figure 3). The free TIMB, TPA, IPA, and TPDC molecules display primary emission peaks at approximately 450, 385, 360, and 340 nm, respectively. As depicted in Figure 3a, **NTOU-12a** features a broad emission band centered at 390 nm that significantly overlaps with the emission profiles of the free nitrogen- and oxygen-donor ligands, suggesting that its luminescence originates primarily from the TIMB and TPA linkers. Furthermore, compared to the emission profiles of the free TIMB, IPA, and TPDC ligands, **NTOU-12b** and **NTOU-12c** exhibit distinct blue- and red-shifted emissions, respectively (Figure 3b,c). These shifts are likely attributable to complex structural features, intra-ligand, and/or ligand-to-ligand charge transfer processes. The nature, spatial arrangements, and coordination environments of metal ions and ligands serve as the key factors for modulating the luminescence properties of those materials.^27^ One interesting example of two MOF compounds demonstrated the emitting color from blue to purple/blue emission by controlling the local ligand environments.^28^ The CIE chromaticity coordinates also confirm that **NTOU-12c** exhibits green emission under 254 nm excitation (0.31, 0.44), while the title compounds display blue luminescence under other irradiation conditions. The coordinates (0.24, 0.23), (0.19, 0.19), (0.22, 0.22), (0.24, 0.27), and (0.23, 0.222) fall within the blue-emitting region, whereas the (0.31, 0.44) coordinate is located in the green region (Figure S14). Inspired by the RGB concept, we explored tuning the emission colors by adsorbing lanthanide (Ln) ions onto the title compounds. As detailed in the Experimental Section, fine powder samples were treated with lanthanide solutions of varying Eu^3+^/Tb^3+^ molar ratios to systematically explore the emission color tuning. The pristine compounds and the Ln-adsorbed products exhibited a wide range of emission colors, including blue, red, pink, yellow, and green (Figures S15–S17 and Figure 4). The photoluminescence spectra (Figures S15a, S16a, and S17a) revealed the characteristic emission bands of the lanthanide ions adsorbed onto the host structures: for compounds contained Eu^3+^ ions (under excitation at 254 nm), ^5^D_0_ → ^7^F_1_ (590 nm), ^5^D_0_ → ^7^F_2_ (619 nm), ^5^D_0_ → ^7^F_3_ (650 nm) and ^5^D_0_ → ^7^F_4_ (700 nm) transitions; for Tb^3+^-adsorbed samples (under excitation at 300 nm), ^5^D_4_ → ^7^F_6_ (490 nm), ^5^D_4_ → ^7^F_5_ (545 nm), ^5^D_4_ → ^7^F_4_ (590 nm) and ^5^D_4_ → ^7^F_3_ (620 nm). The intensity of ^5^D_0_ →^7^F_2_ electric dipole (ED) transition larger than that of the ^5^D_0_ →^7^F_1_ magnetic dipole (MD) implied a lack of inversion symmetry at the Eu^3+^ sites. This non-centrosymmetric coordination environment is consistent with interactions between the adsorbed Ln^3+^ species and the chemically heterogeneous host environment.^29,30^ These observations suggest that Ln^3+^ adsorption is influenced by the structural and chemical characteristics of the host materials. The mixed Eu^3+^/Tb^3+^-adsorbed compounds exhibited the characteristic emission bands of both ions, interacting with the host matrix to produce various luminescent properties.

**Figure 3:**
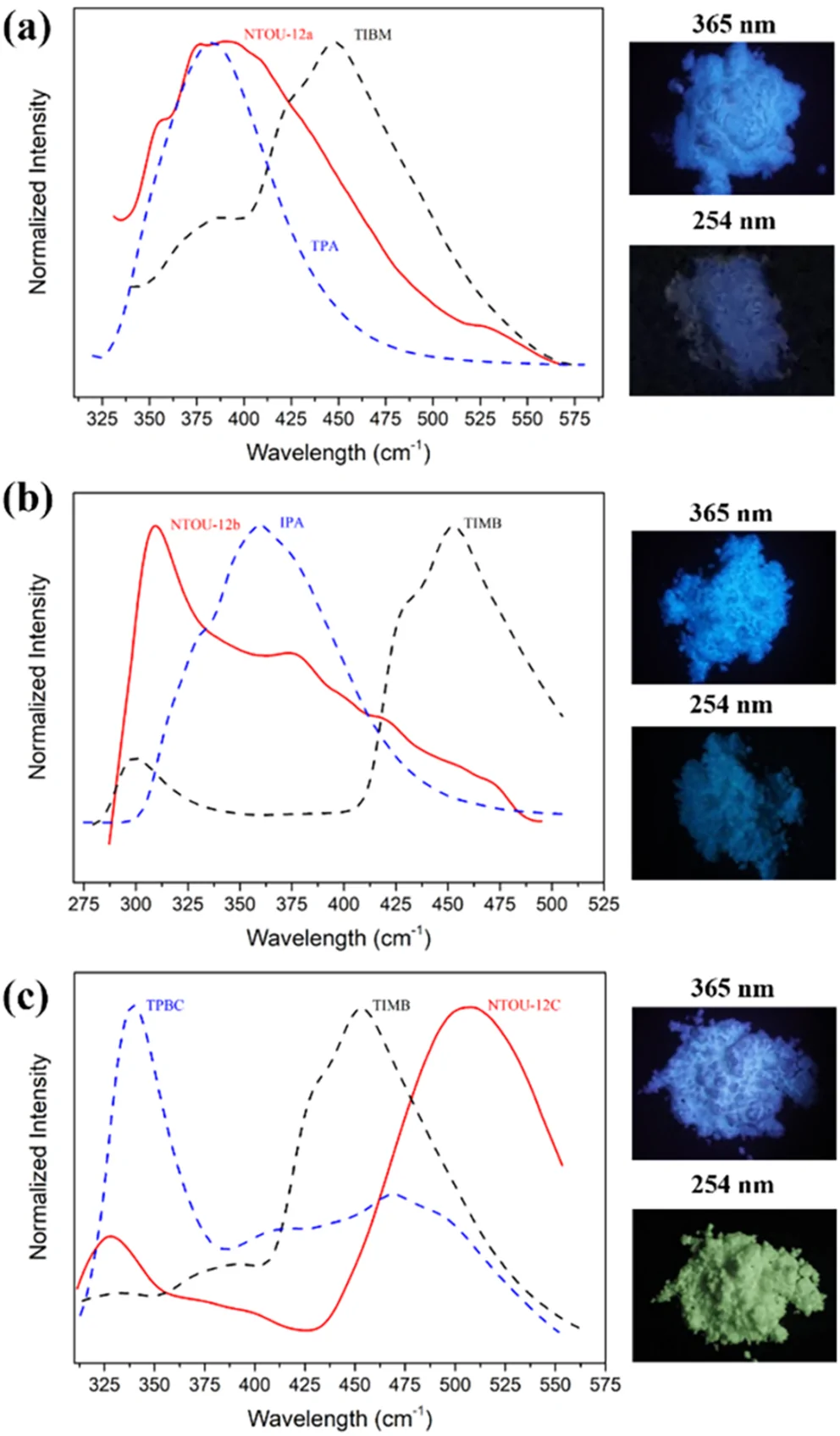
Solid-state emission spectra of the title compounds and their respective free organic ligands at room temperature: (a) **NTOU-12a**, (b) **NTOU-12b**, and (c) **NTOU-12c**. The corresponding optical images of the powder samples under handheld UV lamps under 365 and 254 nm are shown as insets.

**Figure 4:**
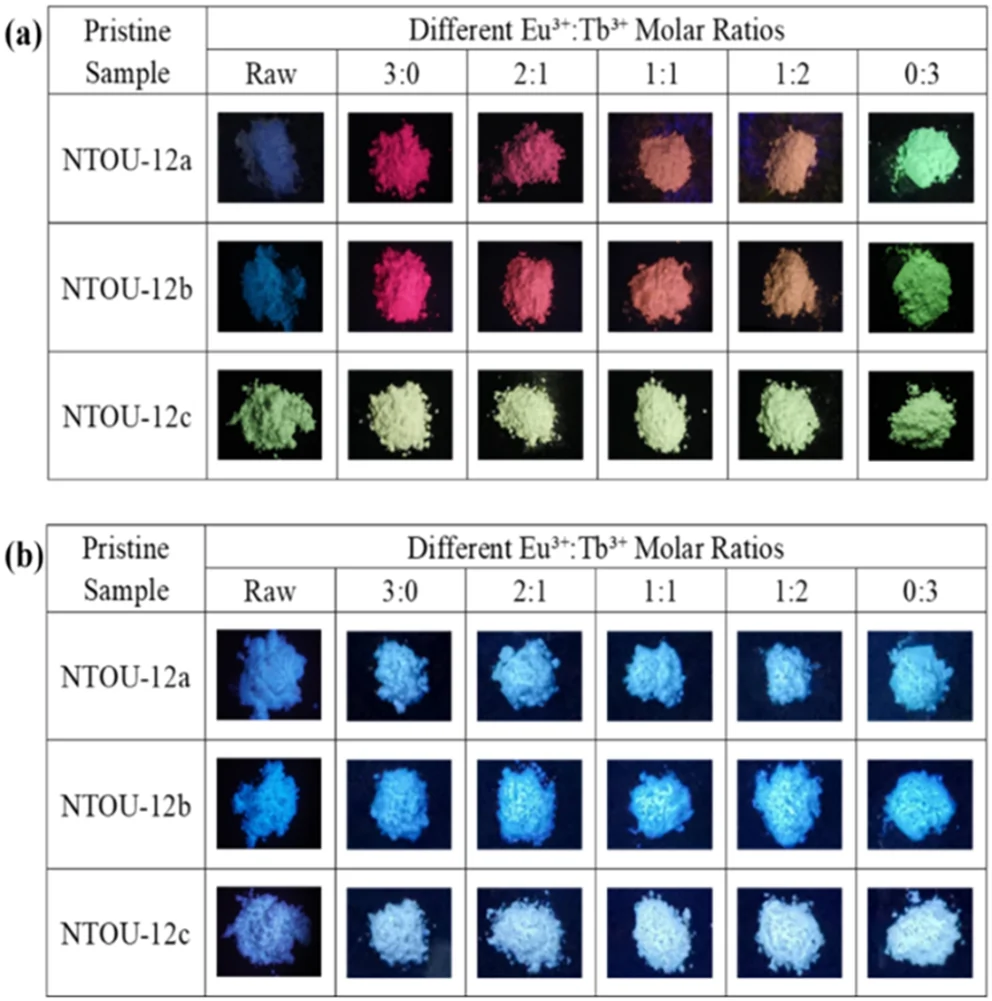
Luminescence properties of the title compounds with varying Eu^3+^/Tb^3+^ molar ratios under the excitation at (a) 254 nm and (b) 365 nm.

The CIE results are summarized in Figure S18 and Table S1 for the title compounds containing different ratios of Eu^3+^ and Tb^3+^ cations. In an effort to achieve white-light emission, the powder samples were treated with 5 mL of a 12 mM lanthanide water/ethanol solution (Eu^3+^/Tb^3+^ = 1/4) under identical reaction conditions. Interestingly, the Ln-adsorbed samples exhibited blue emission under a handheld UV lamp at 365 nm. In contrast, under 254 nm excitation, **NTOU-12a**, **-12b**, and **-12c** displayed white, yellow, and green luminescence, respectively (Figure 5). Their corresponding CIE coordinates (Figure S19) were recorded as (0.32, 0.34) and (0.20, 0.20); (0.33, 0.35) and (0.21, 0.20); and (0.32, 0.42) and (0.21, 0.20). ICP-OES analysis reveals that the Eu^3+^ uptake of **NTOU-12c** (3.47 mg/g) was 27.1% and 41.5% lower than those of **NTOU-12a** (4.76 mg/g) and **NTOU-12b** (5.93 mg/g), respectively. Similarly, the Tb^3+^ uptake of **NTOU-12c** (6.93 mg/g) was 31.5% and 41.4% lower than those of **NTOU-12a** (10.12 mg/g) and **NTOU-12b** (11.82 mg/g), respectively (Table S2). The calculated adsorbed Eu^3+^/Tb^3+^ molar ratios are approximately 1/2.0, 1/1.9, and 1/1.9 for **NTOU-12a**, **NTOU-12b**, and **NTOU-12c**, respectively. Attempts to identify the Ln^3+^ adsorption sites of the three title compounds via SCXRD analyses were unsuccessful, as the single crystals were no longer suitable for structural analysis after being soaked in the aqueous ethanol solutions containing Ln^3+^ ions. Based on luminescence, ICP, and PLATON calculations, the Ln^3+^ adsorbed onto the title compounds may be stemmed from the synergistic effect of surface confinement and these altered electronic properties. While the thiophene sulfur atom in TPDC does not directly coordinate to the lanthanide ions, its presence significantly alters the spatial distribution of Lewis basic sites and the polarity of the crystal surface. This modified local microenvironment may be less favorable for the adsorption of hard lanthanide cations (Eu^3+^ and Tb^3+^), consistent with the observed lower uptake of **NTOU-12c** and the proposed HSAB-based interpretation. Recently, the rational tuning of structural microenvironments has been demonstrated as an effective strategy to modulate chemical and physical properties of coordination polymers, such as host-guest affinities and luminescent behaviors.^27,31^ A two-fold interpenetrated metal–organic framework featured a nanotrap structure for extracting lanthanide elements from wastewater via its carboxyl groups and triazole nitrogen atoms.^32^ The CPM-66 MOF series with the 6-coordinated rare-earth ions and 6-connected trimers demonstrated a restricted coordination environment, improving the separation selectivity for Ln elements.^33^ The spatial distribution of Lewis basic sites, steric confinement, and the electronic properties of the linkers in materials also impact both the adsorption efficiency of guest ions and the subsequent ligand-to-metal energy transfer.^34–36^ For the **NTOU-12** series, maintaining a similar structural framework while varying the coordinating ligands successfully establishes a highly adaptable synthetic platform. The distinct microenvironmental structures within these frameworks not only govern the varied Ln^3+^ uptake capacities but also selectively tune the photoluminescence profiles. This ligand-substitution approach also might provide a rational framework for designing multifunctional materials with tunable luminescence and selective lanthanide capture.

**Figure 5:**
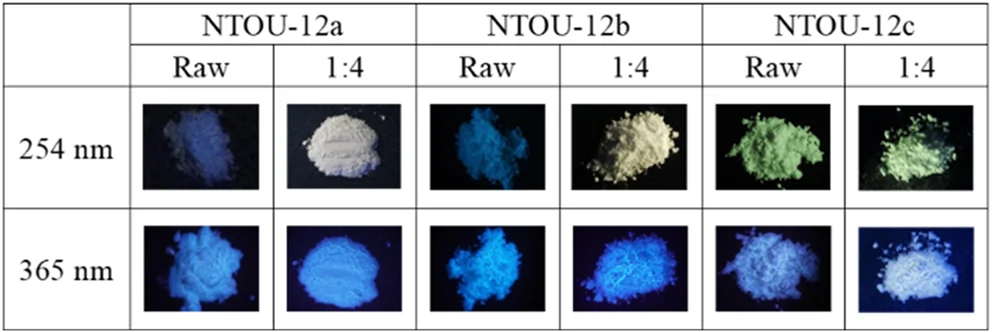
Luminescence properties of the title compounds with a Eu^3+^/Tb^3+^ molar ratio of 1/4 upon excitation at 254 and 365 nm.

## Conclusion

In summary, a series of robust organic-inorganic hybrid zincophosphites (**NTOU-12a**, **-12b**, and **-12c**) were successfully obtained through direct synthesis incorporating discrete dicarboxylate ligands. SCXRD analyses demonstrate that varying the choice of the dicarboxylate component in the initial reaction mixtures allows us to synthesize similar framework topology but with different microenvironmental structures. In comparison with **NTOU-12a**, the geometry of the IPA ligand yields an expanded architecture in **NTOU-12b** capable of hosting lattice water molecules, whereas the assembly of **NTOU-12c** with the sulfur-containing TPDC linker deliberately alters the local coordination environment. By leveraging Hard-Soft Acid–Base (HSAB) principles during the initial crystallization process, the modified microenvironment of **NTOU-12c** influences its interactions with adsorbed Ln^3+^ species, leading to a 27–42% reduction in Eu^3+^ adsorption and a 32–41% reduction in Tb^3+^ uptake compared to **NTOU-12a** and **-12b** with other oxygen-donor analogs. This tailored host-guest interplay demonstrates the structural adaptability of the **NTOU-12** topology and enables the fine-tuning of multi-color emissions, culminating in white-light luminescence. Beyond the specific properties of these compounds, this work provides a rational design strategy. The ability to integrate structural stability with targeted chemical modulation (via HSAB principles) offers a solid foundation for developing materials targeted at selective lanthanide separation and the fabrication of optical devices.

## Supplementary Material



## Notes

Coordination environments for zinc and phosphorus atoms and organic ligands, structural stability, luminescence, and ICP-OES measurements (PDF)
