# Supplementary material for: Ligand Substitution in Three Organic–Inorganic Hybrid Zincophosphite Analogs: Modulating Lanthanide Affinity and White-Light Emission via Hard–Soft Acid–Base Interplay
Source: Inorg Chem. 2026 Sep 12;65(38):22820–7. doi: 10.1021/acs.inorgchem.6c04380 (PMC13625946; doi:10.1021/acs.inorgchem.6c04380)
Supplement: Supplementary file 1 [file ic6c04380_si_001.pdf]

Supporting Information

Ligand Substitution in Three Organic-Inorganic Hybrid Zincophosphite Analogs: Modulating Lanthanide Affinity and White-Light Emission via Hard-Soft Acid-Base Interplay

Mu-Chien Yin,[a,+] Chih-Feng Yen,[b,+] Tzu-Yuan Sung,[c,+] Ju-Ying Chen,[a] Jui Hsieh,[a] Chun-Ru Hsu,[a] Wei-Cheng Chen,[a] Ming-Der Wu,[d] and Chih-Min Wang*,[a]

[a]Department of Bioscience and Biotechnology, National Taiwan Ocean University, Keelung 202, Taiwan, R.O.C.
[b]Department of Microelectronics Engineering, National Kaohsiung University of Science and Technology, Kaohsiung 811, Taiwan, R.O.C.
[c]Department of Graduate Institute of Aquaculture, National Kaohsiung University of Science and Technology, Kaohsiung 811, Taiwan, R.O.C.
[d]Bioresource Collection and Research Center, Food Industry Research and Development Institute, Hsinchu 300, Taiwan, R.O.C.
[+]These authors contributed equally to this work.

E-mail of corresponding author:
twcmwang@gmail.com; cmwang@ntou.edu.tw (CMWang)

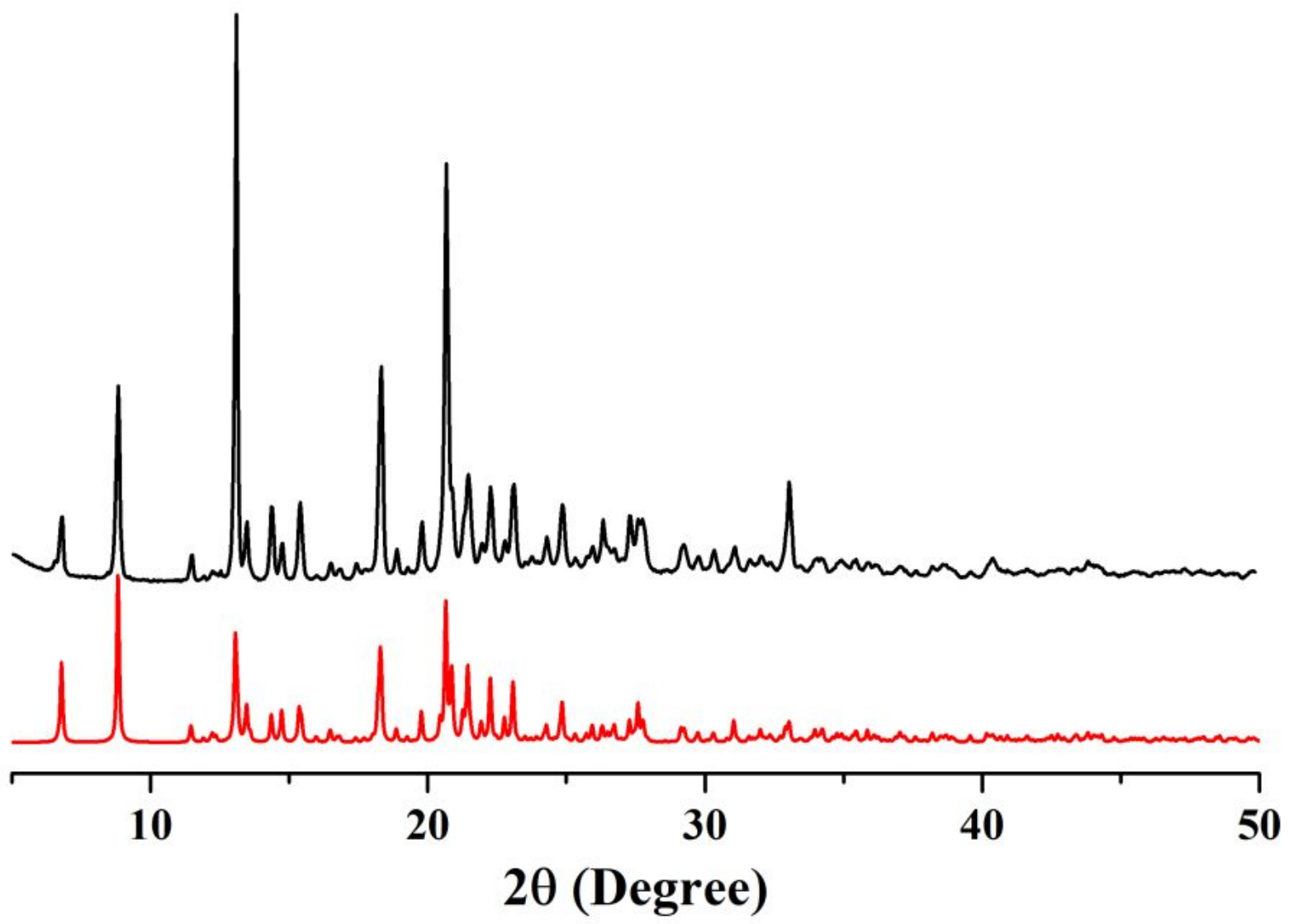

Figure S1. PXRD patterns for **NTOU-12a**. Experimental and simulated patterns are shown in black and red, respectively.

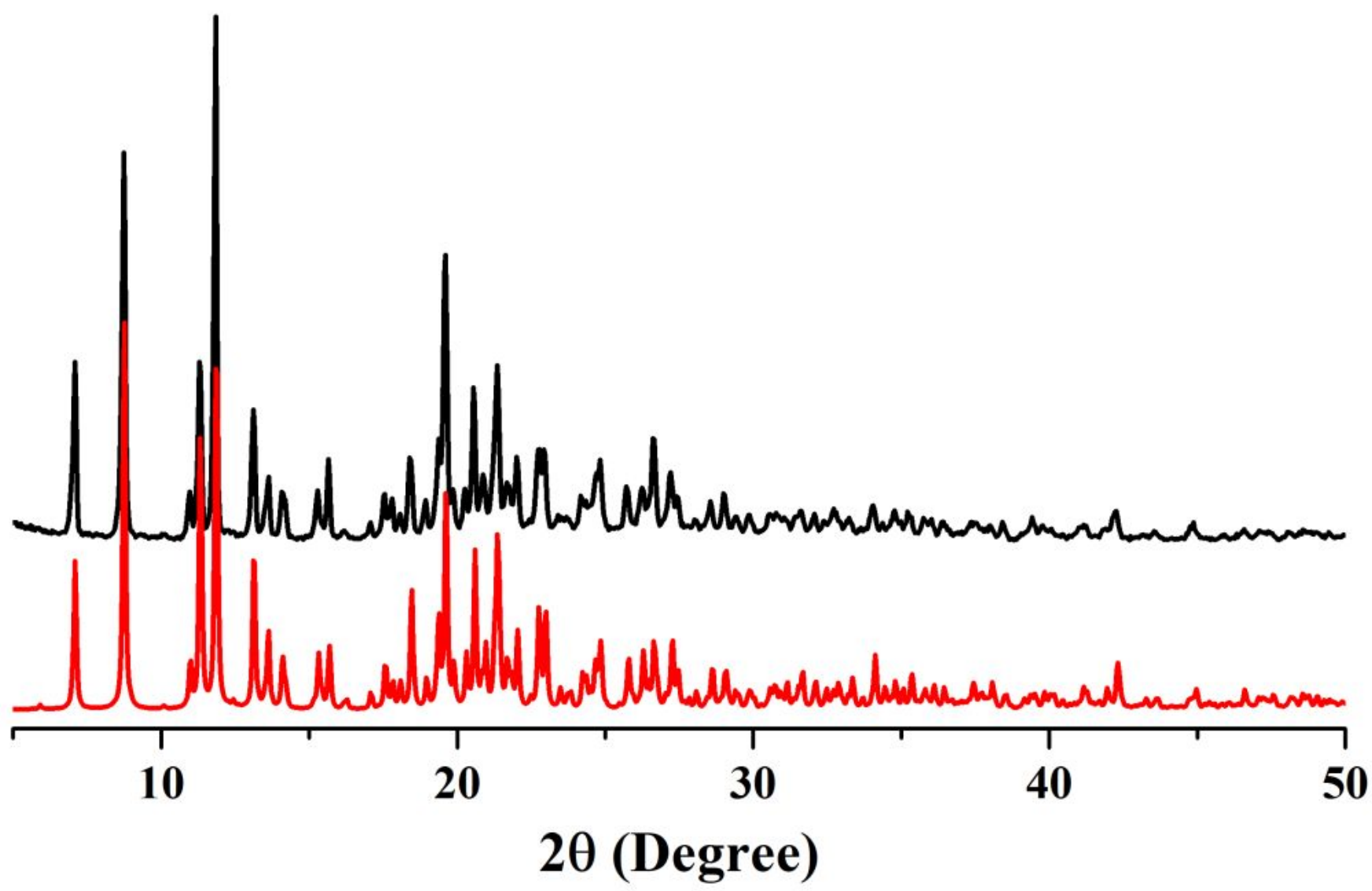

Figure S2. PXRD patterns for **NTOU-12b**. Experimental and simulated patterns are shown in black and red, respectively.

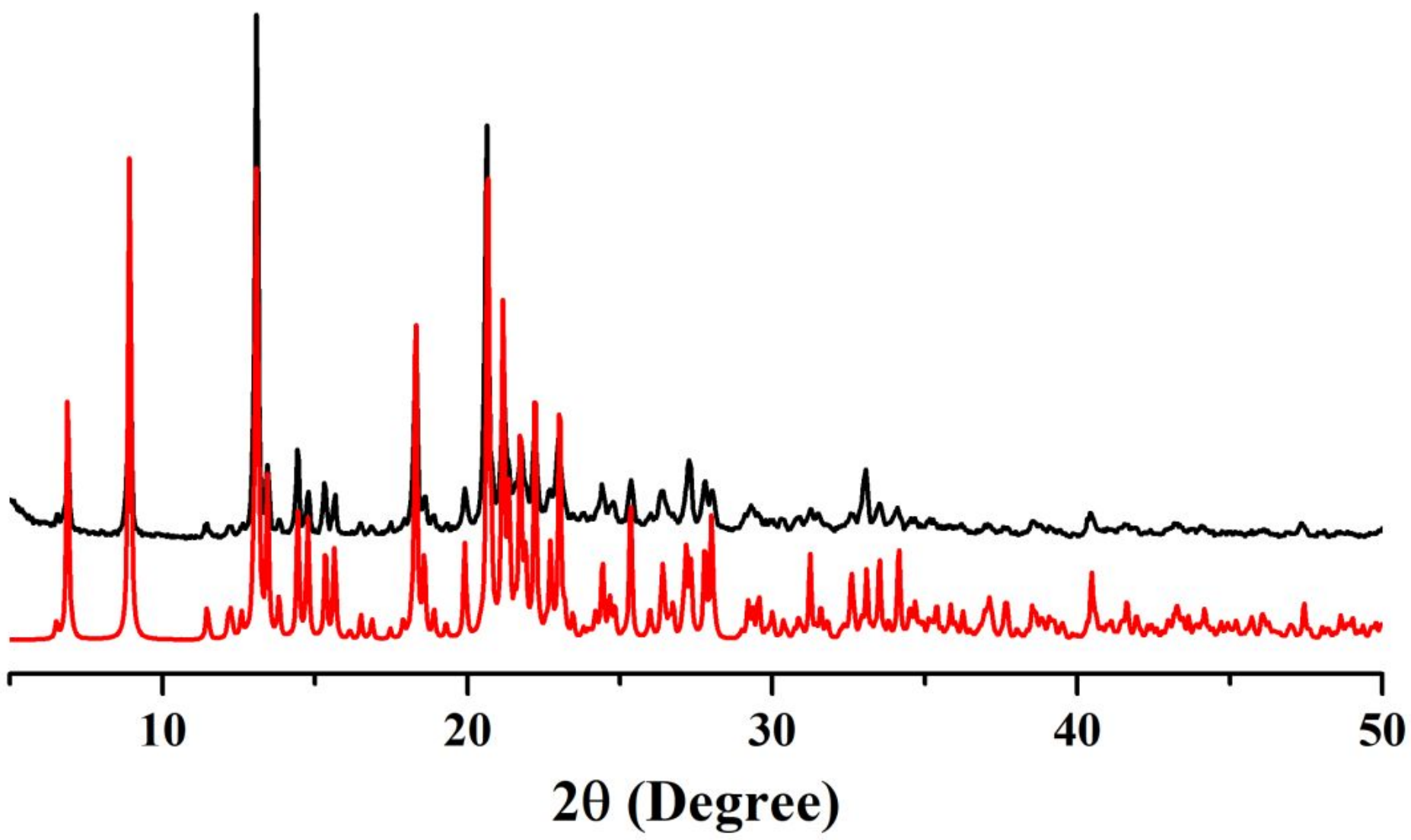

Figure S3. PXRD patterns for **NTOU-12c**. Experimental and simulated patterns are shown in black and red, respectively.

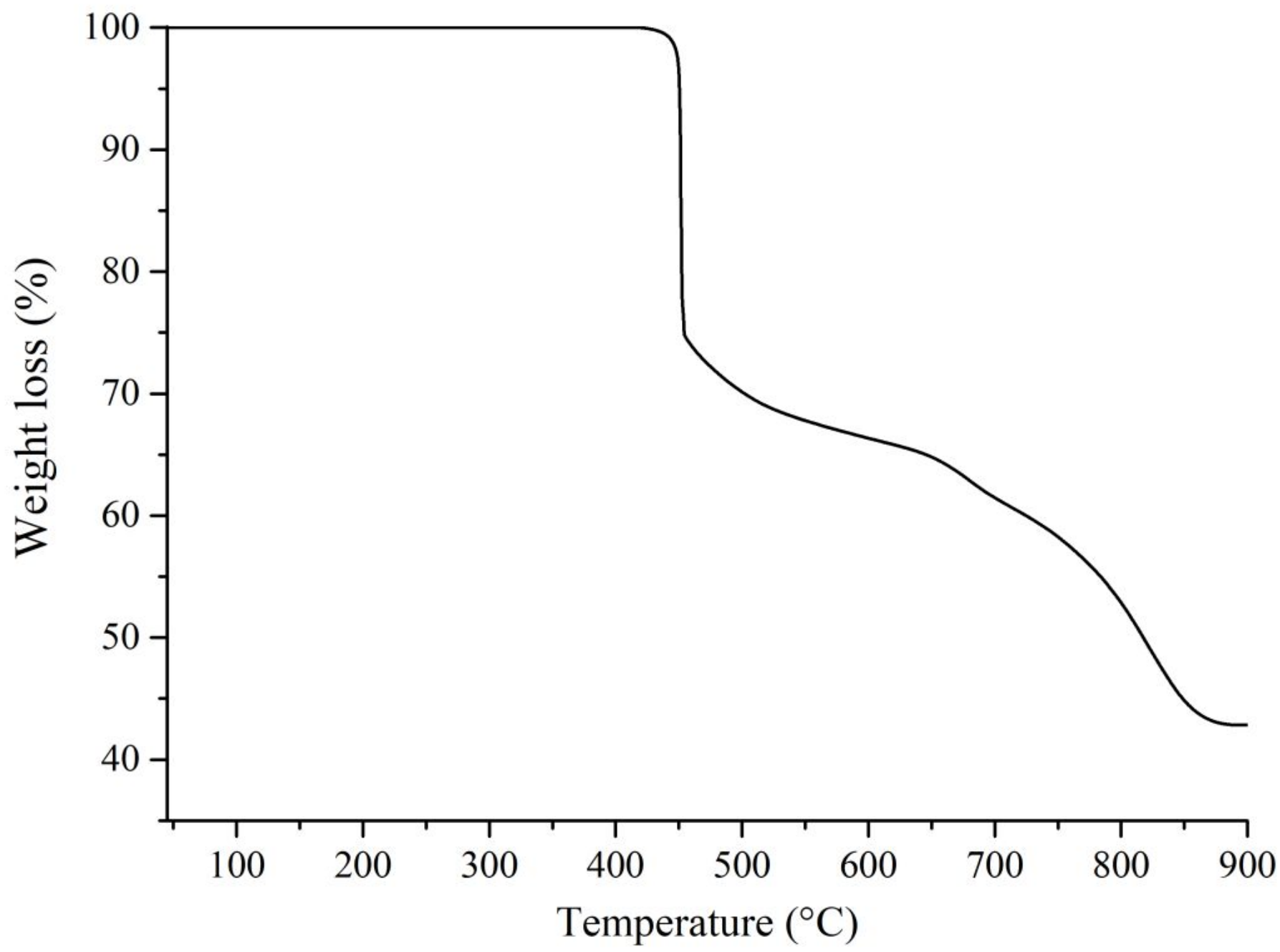

Figure S4. TGA curve in flowing O_2 gas for **NTOU-12a**.

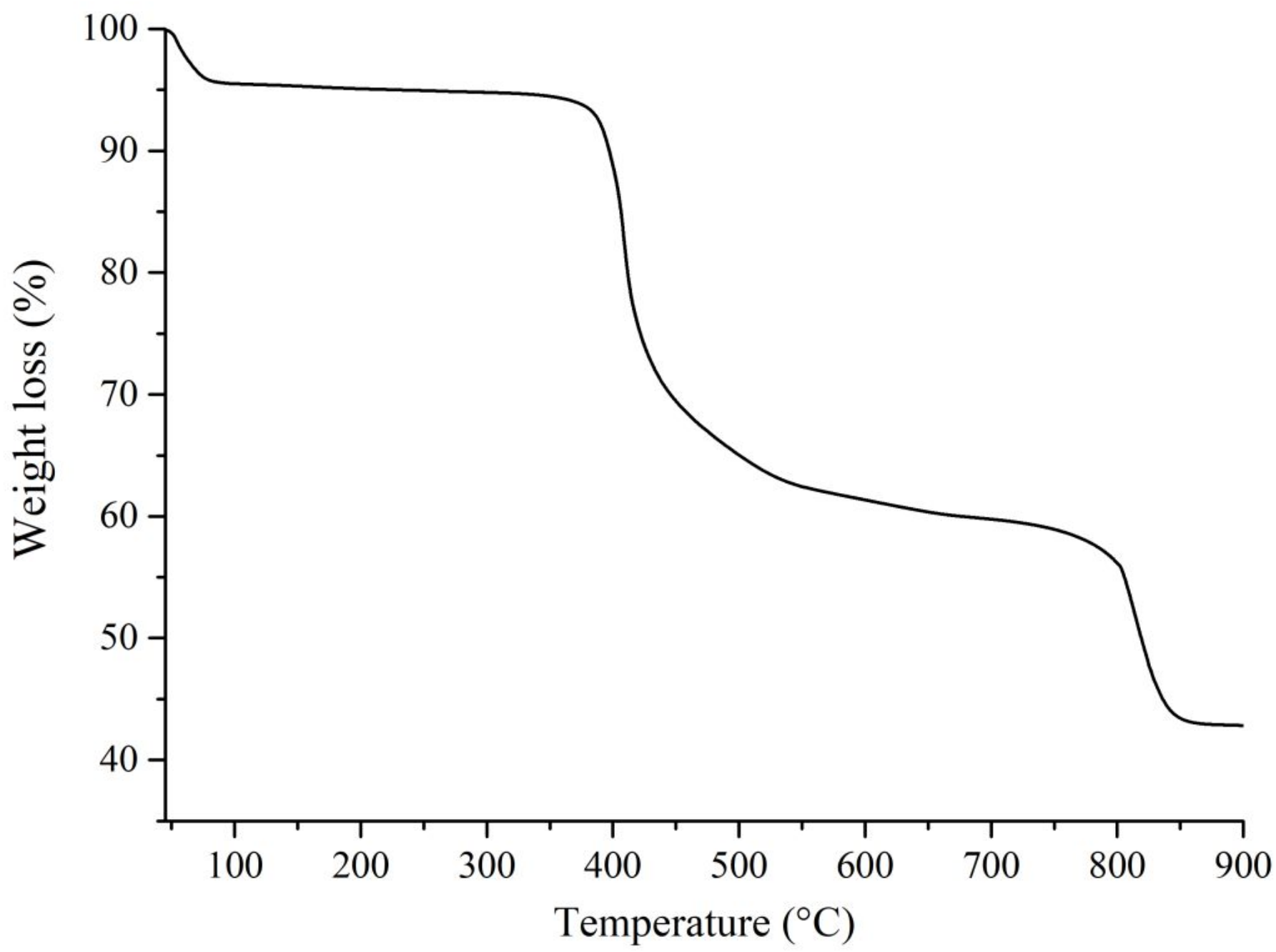

Figure S5. TGA curve in flowing O_2 gas for **NTOU-12b**.

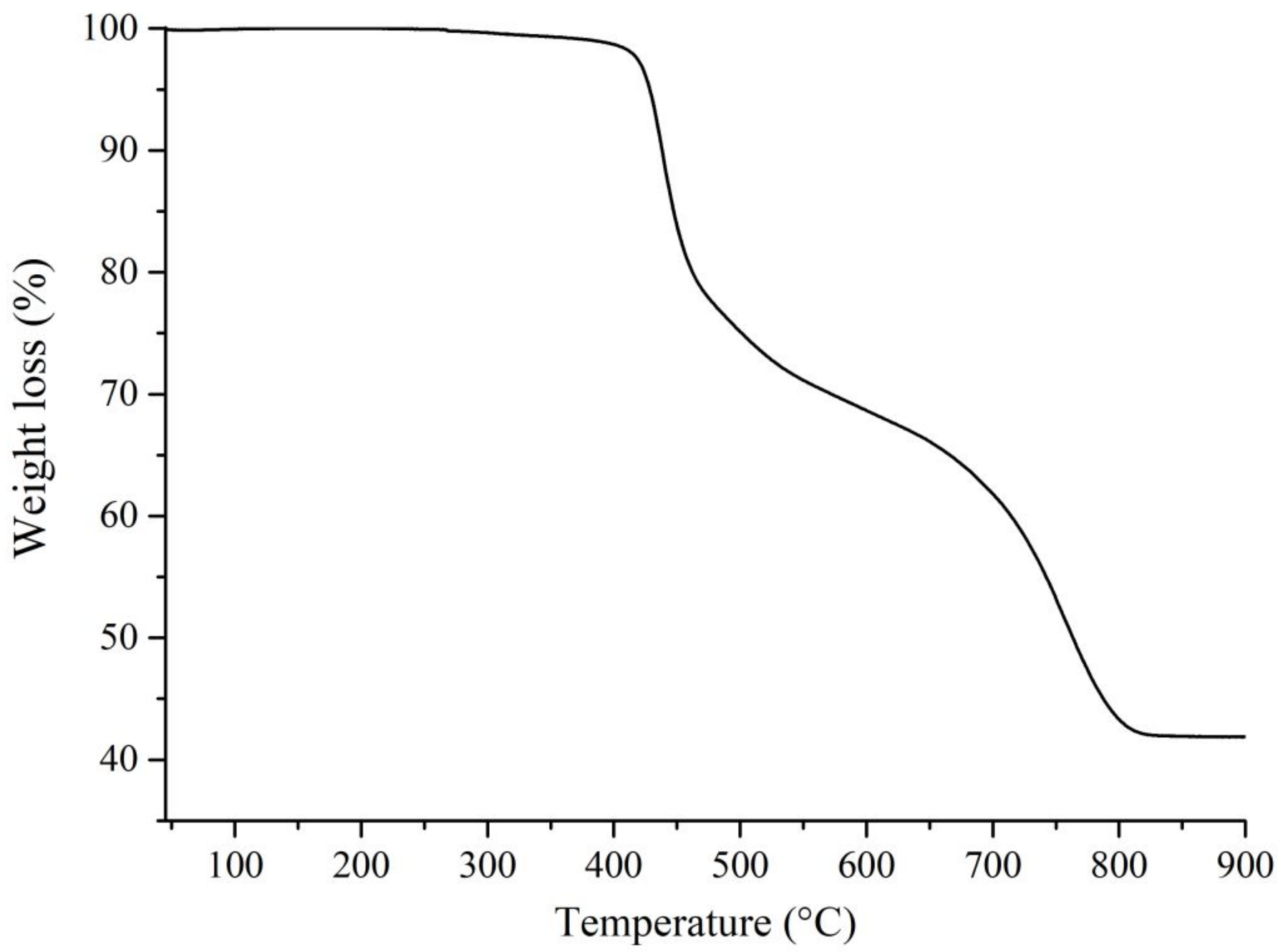

Figure S6. TGA curve in flowing O_2 gas for **NTOU-12c**.

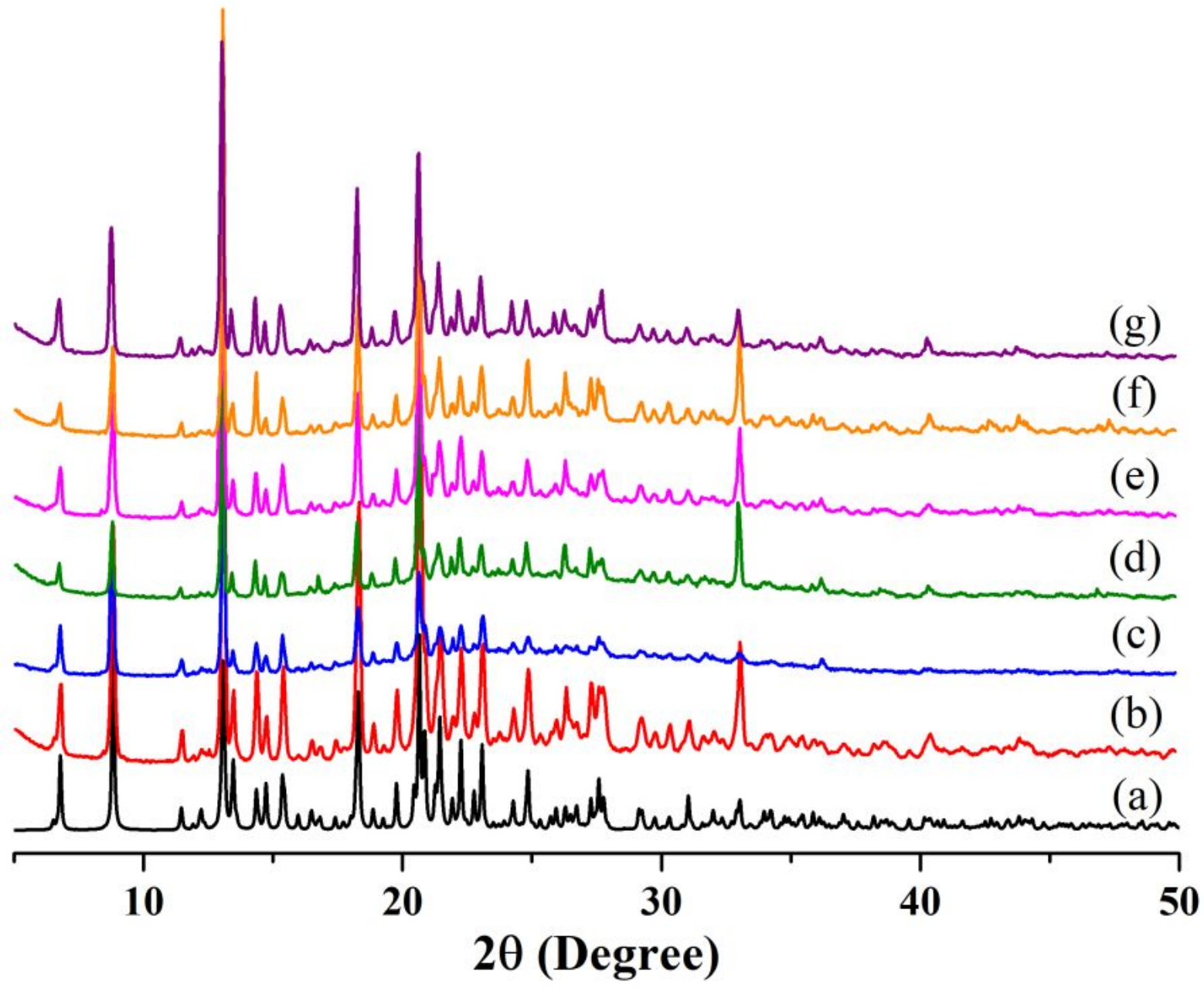

Figure S7. X-ray powder diffraction patterns of **NTOU-12a** for thermal stability studies. (a) Simulated, (b) as-synthesized, (c) holding for 30 mins at 200 °C, (d) 250 °C, (e) 300 °C, (f) 350 °C, and (g) 400 °C.

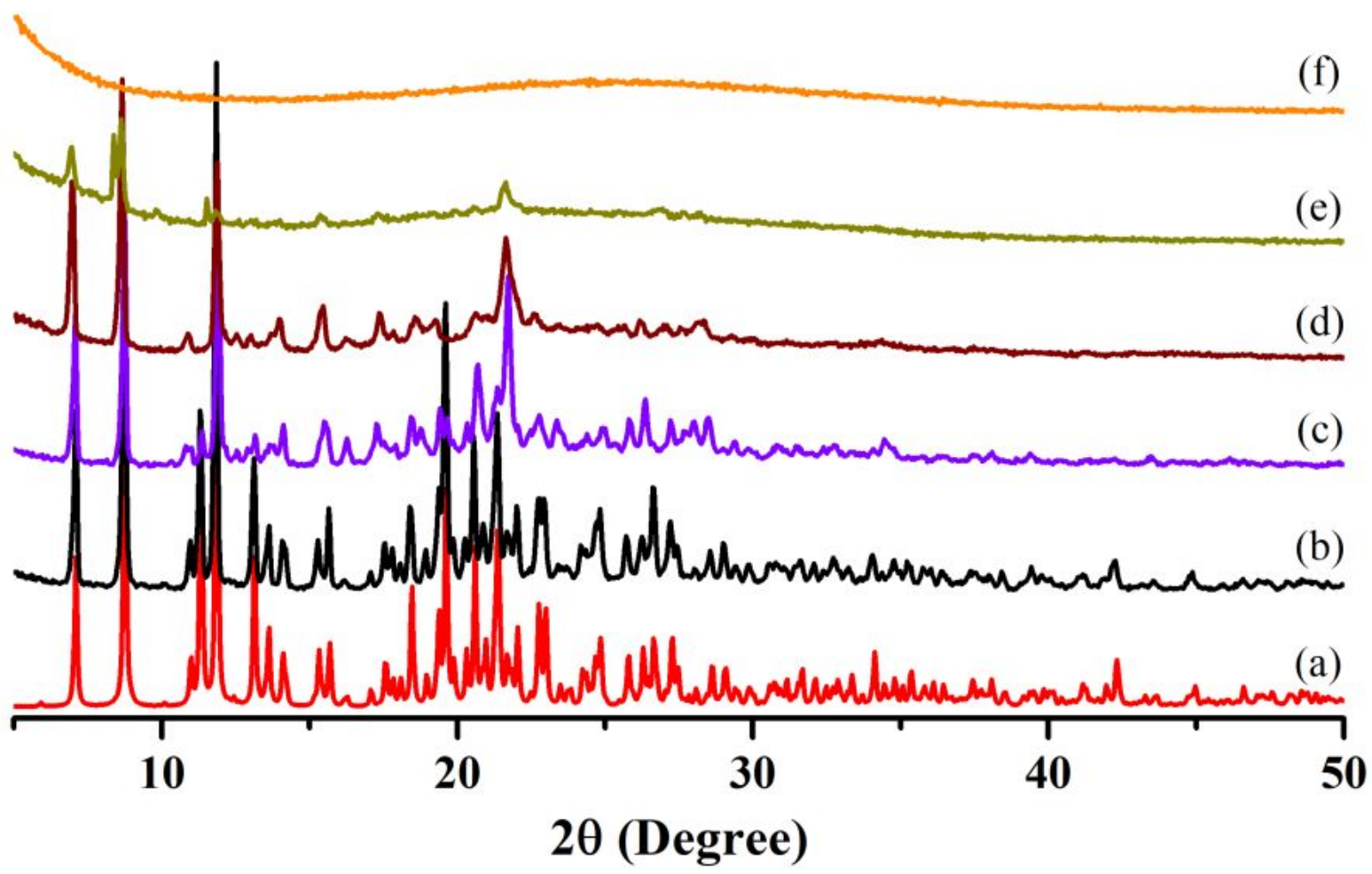

Figure S8. X-ray powder diffraction patterns of **NTOU-12b** for thermal stability studies. (a) Simulated, (b) as-synthesized, (c) holding for 30 mins at 250 °C, (d) 300 °C, (e) 350 °C, and (f) 400 °C.

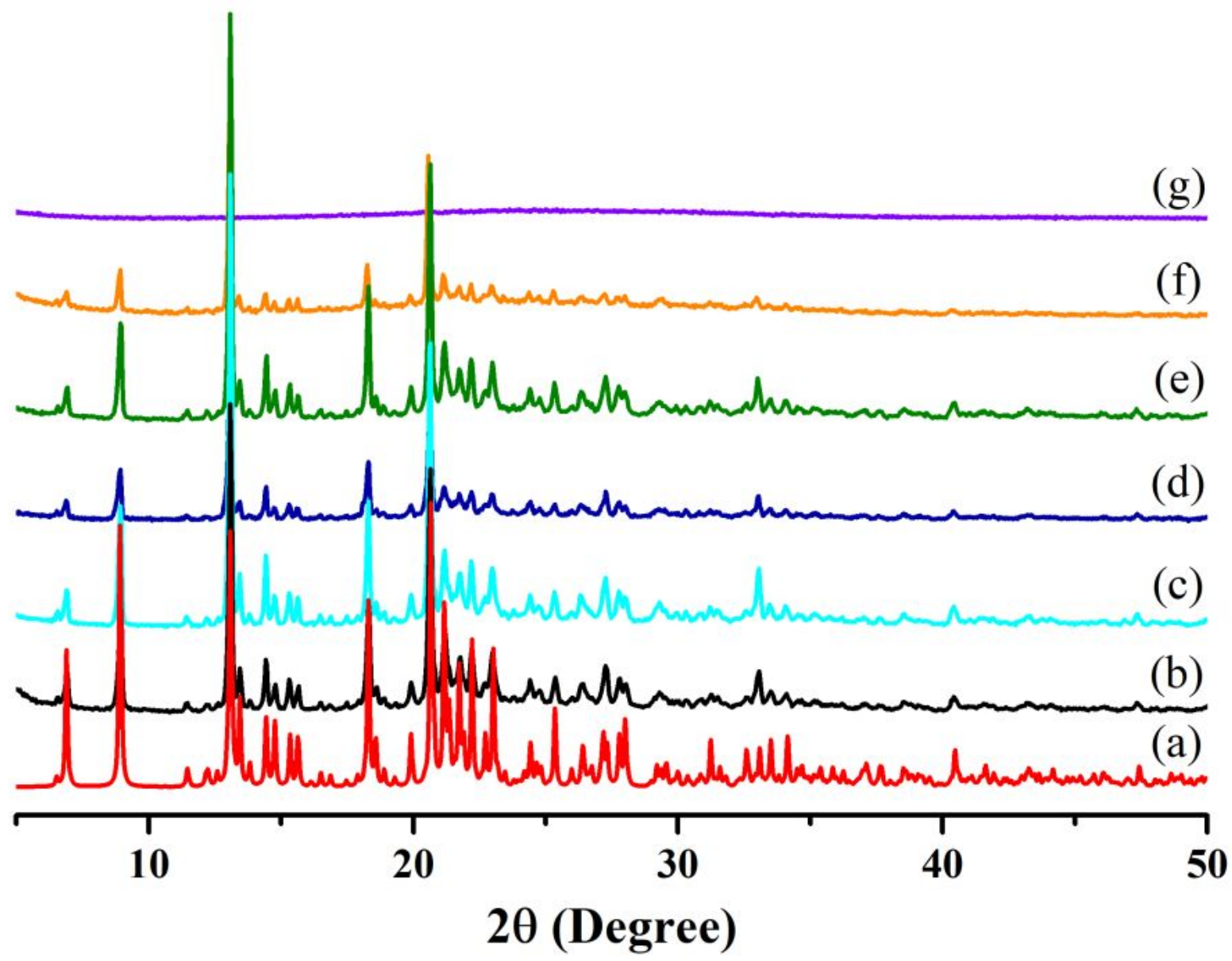

Figure S9. X-ray powder diffraction patterns of **NTOU-12c** for thermal stability studies. (a) Simulated, (b) as-synthesized, (c) holding for 30 mins at 200 °C, (d) 250 °C, (e) 300 °C, (f) 350 °C, and (g) 400 °C.

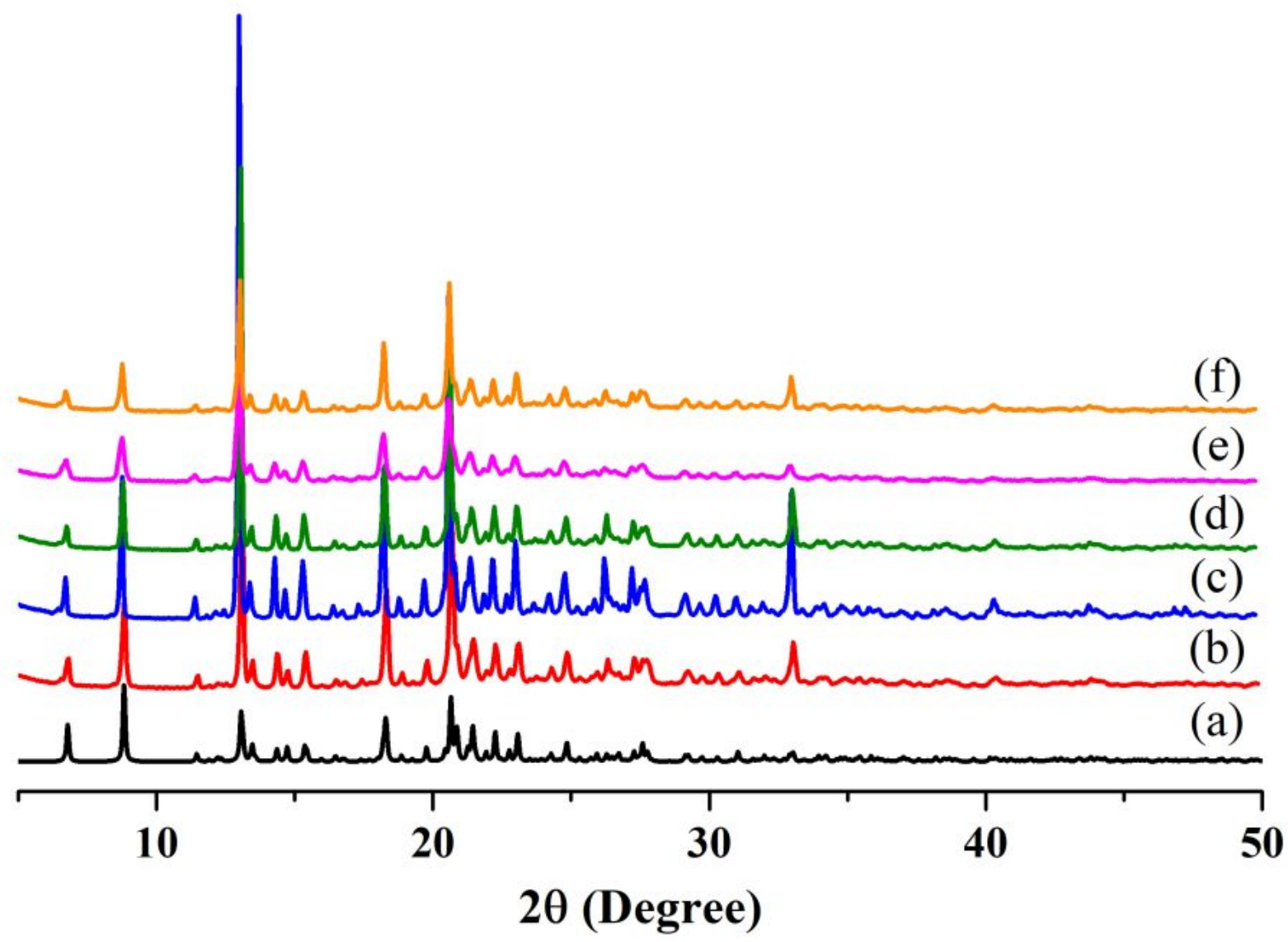

Figure S10. X-ray powder patterns of **NTOU-12a** for chemical stability studies when the powder samples were in contact with aqueous solutions at different pH values for 1 h: simulated (a), as-synthesized (b), pH = 2 (c), 4 (d), 10 (e), 11(f).

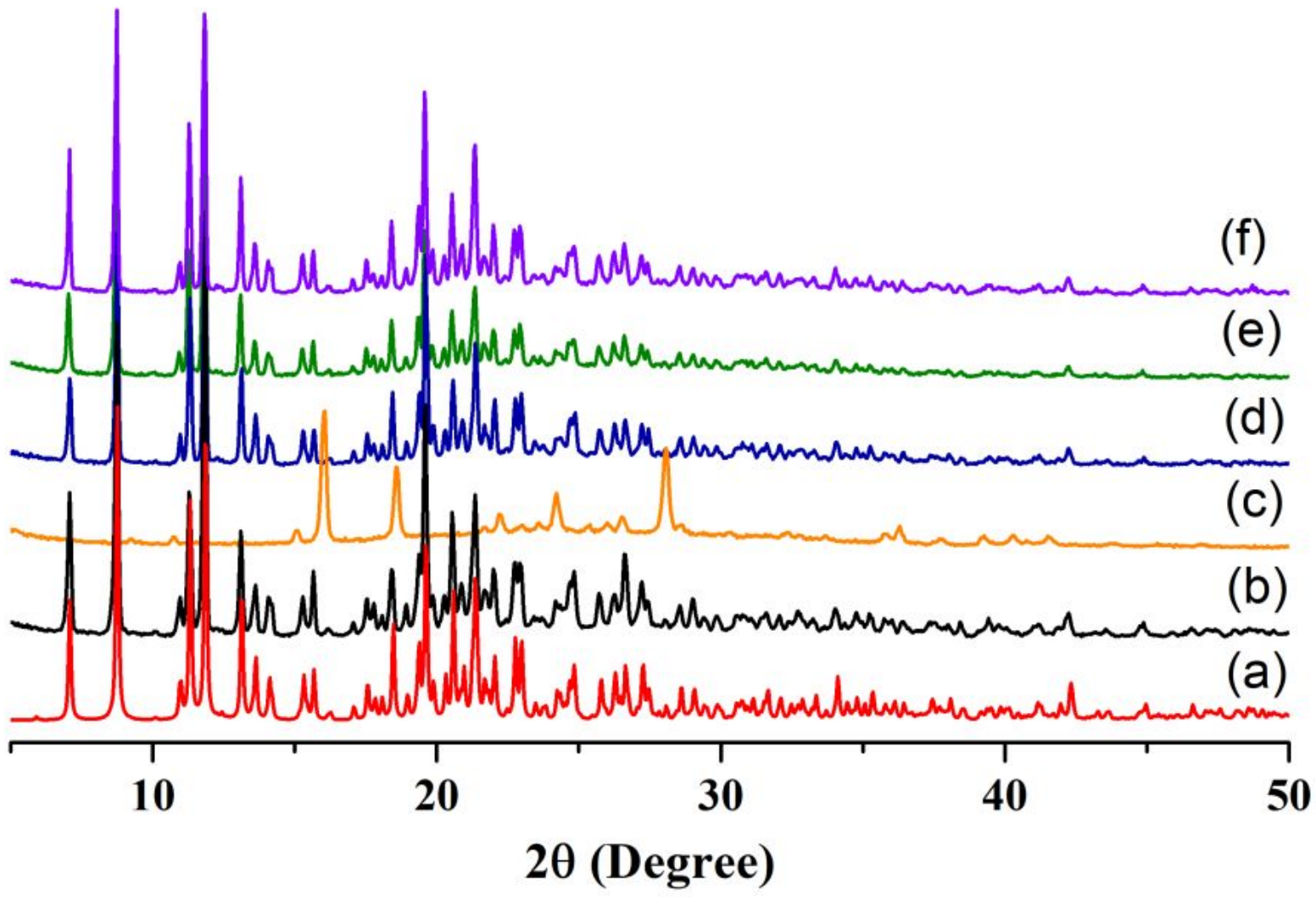

Figure S11. X-ray powder patterns of **NTOU-12b** for chemical stability studies when the powder samples were in contact with aqueous solutions at different pH values for 1 h: simulated (a), as-synthesized (b), 3 (c), 4 (d), 5 (e), and 12 (f).

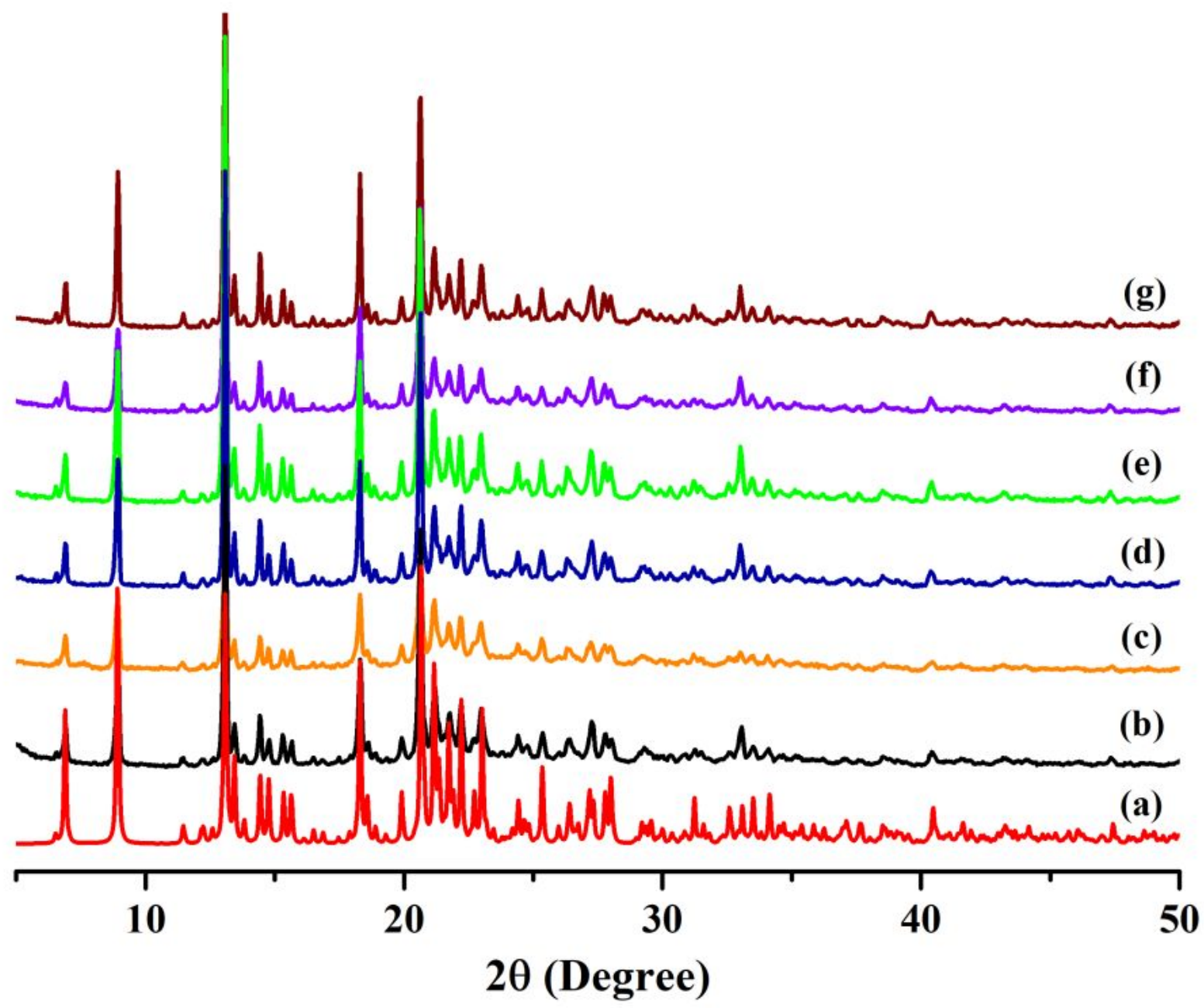

Figure S12. X-ray powder patterns of **NTOU-12c** for chemical stability studies when the powder samples were in contact with aqueous solutions at different pH values for 1 h: simulated (a), as-synthesized (b), pH = 2 (c), 3 (d), 4 (e), 10 (f), and 11 (g).

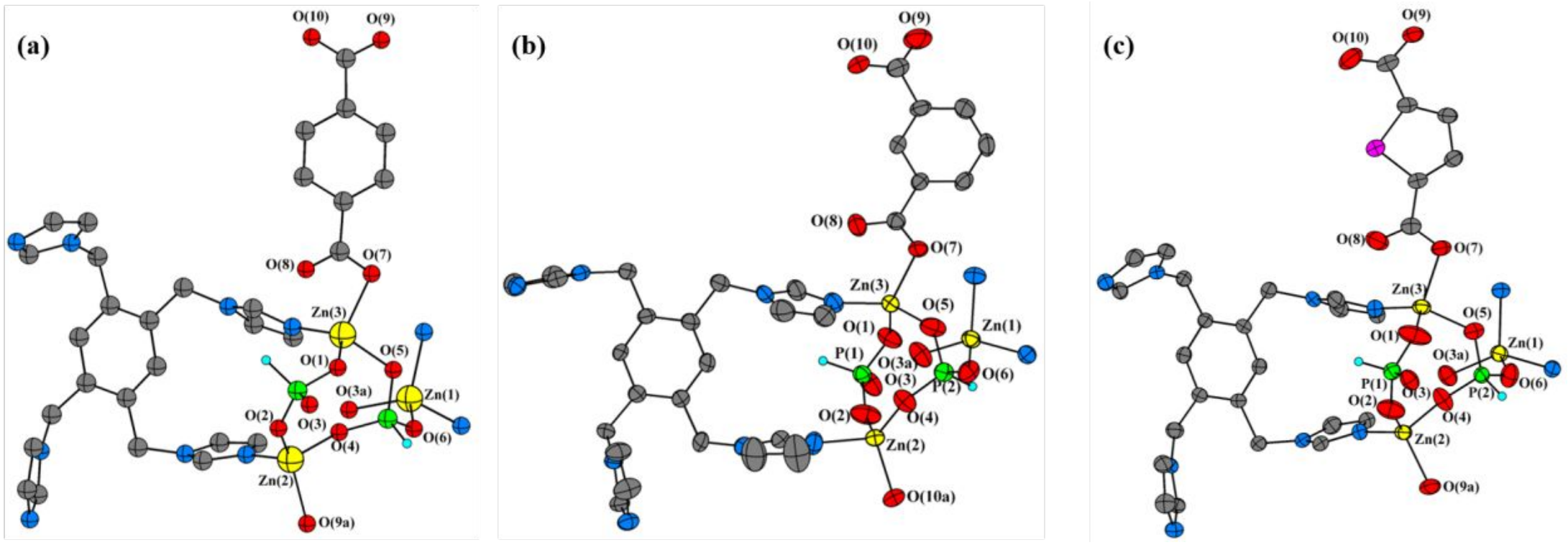

Figure S13. Coordination environment for zinc and phosphorus atoms and organic ligands in **NTOU-12a** (a), **NTOU-12b** (b), **NTOU-12c** (c). Zn, P, N, C, O, and H atoms are indicated in yellow, green, blue, gray, red, and bluish circles, respectively.

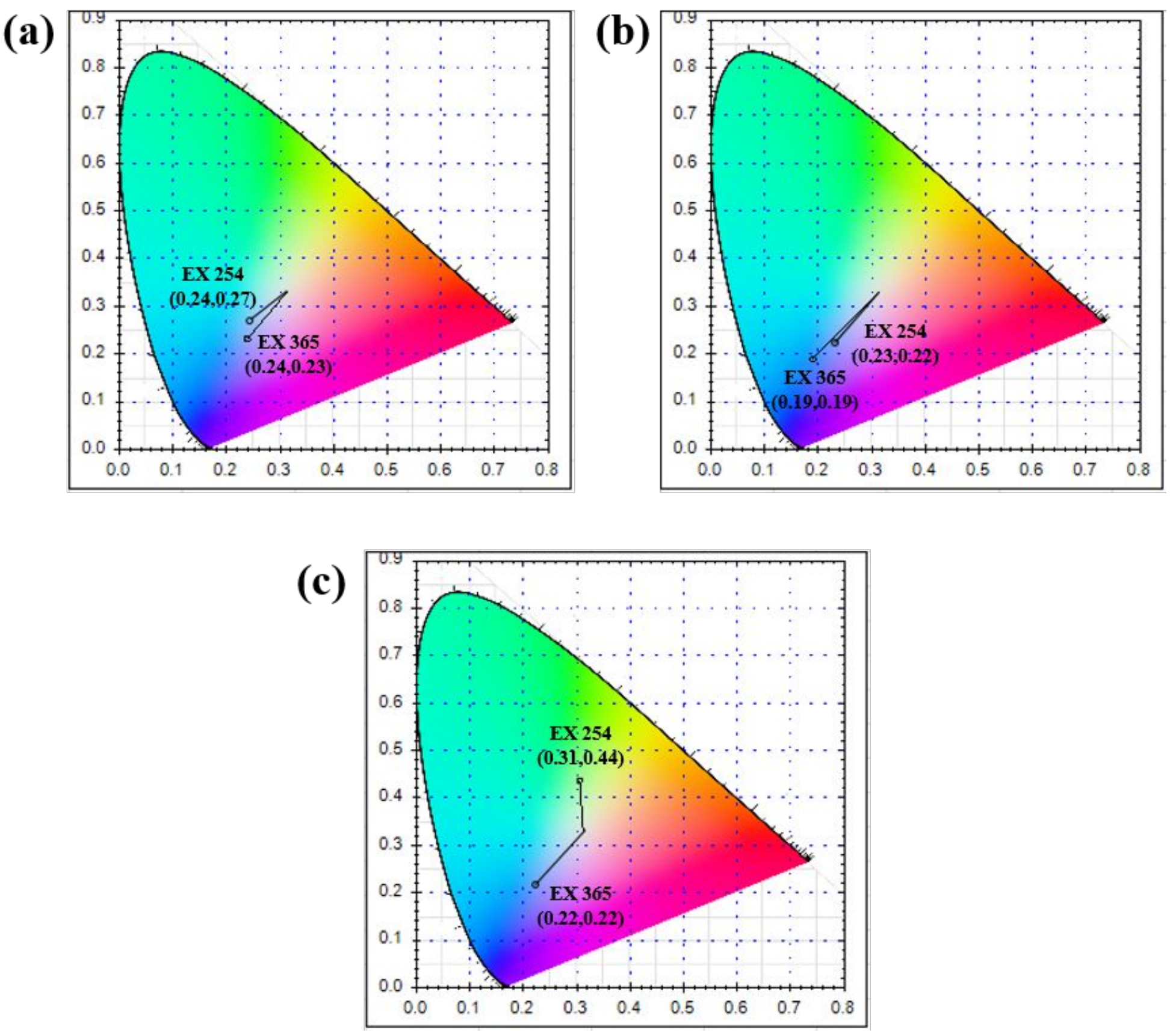

Figure S14. CIE chromaticity diagrams of **NTOU-12a** (a), **NTOU-12b** (b), **and NTOU-12c** (c) under UV light irradiation at 254 nm and 365 nm, respectively.

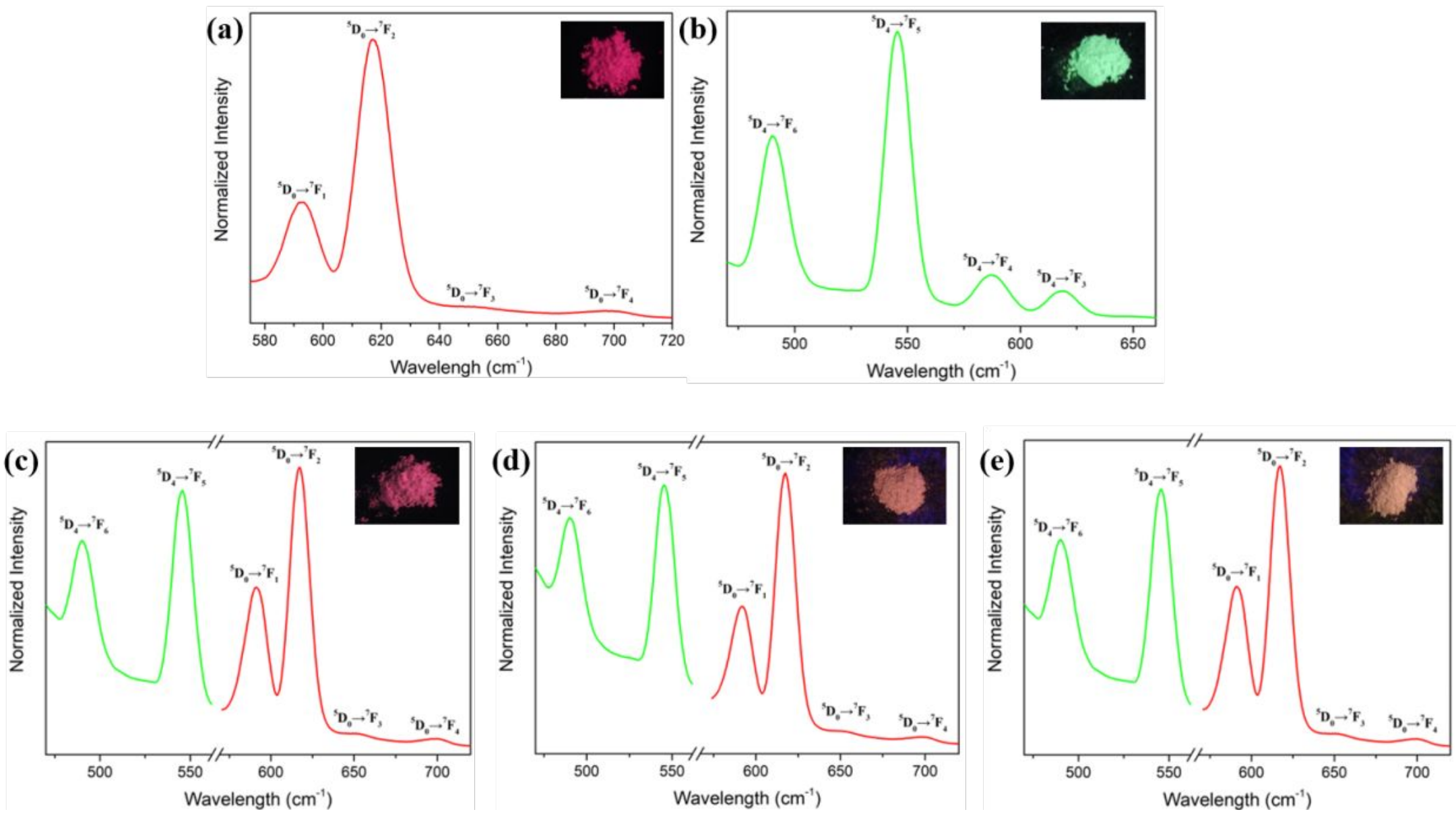

Figure S15. Luminescence properties of compound **NTOU-12a** containing different Eu^{3+}/Tb^{3+} molar ratios: (a) 3/0, (b) 0/3 (c) 2/1, (d) 1/1, and (e) 1/2, under excitation at 254 and/or 300 nm.

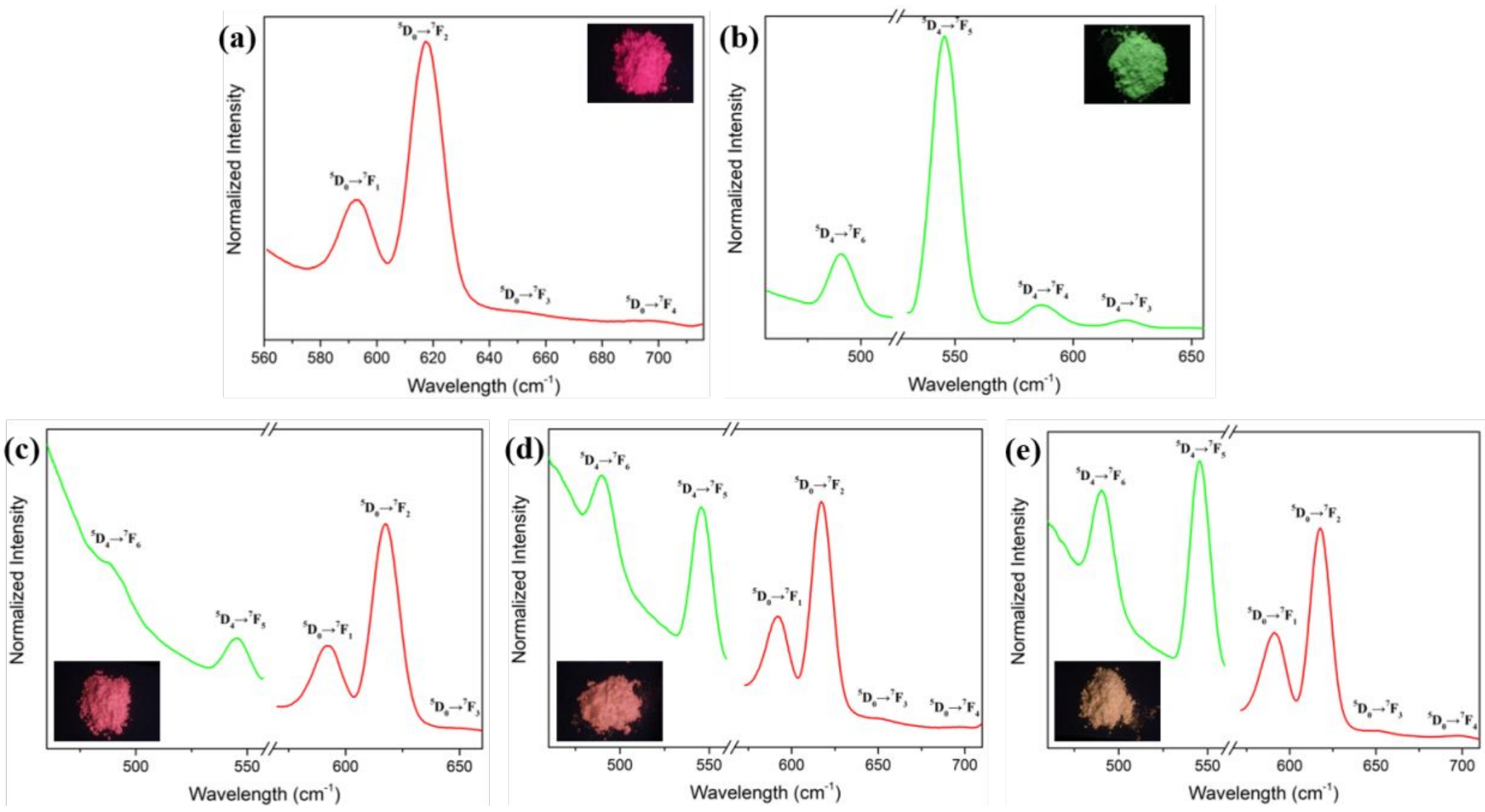

Figure S16. Luminescence properties of compound **NTOU-12b** containing different Eu^{3+}/Tb^{3+} molar ratios: (a) 3/0, (b) 0/3 (c) 2/1, (d) 1/1, and (e) 1/2, under excitation at 250 and/or 300 nm.

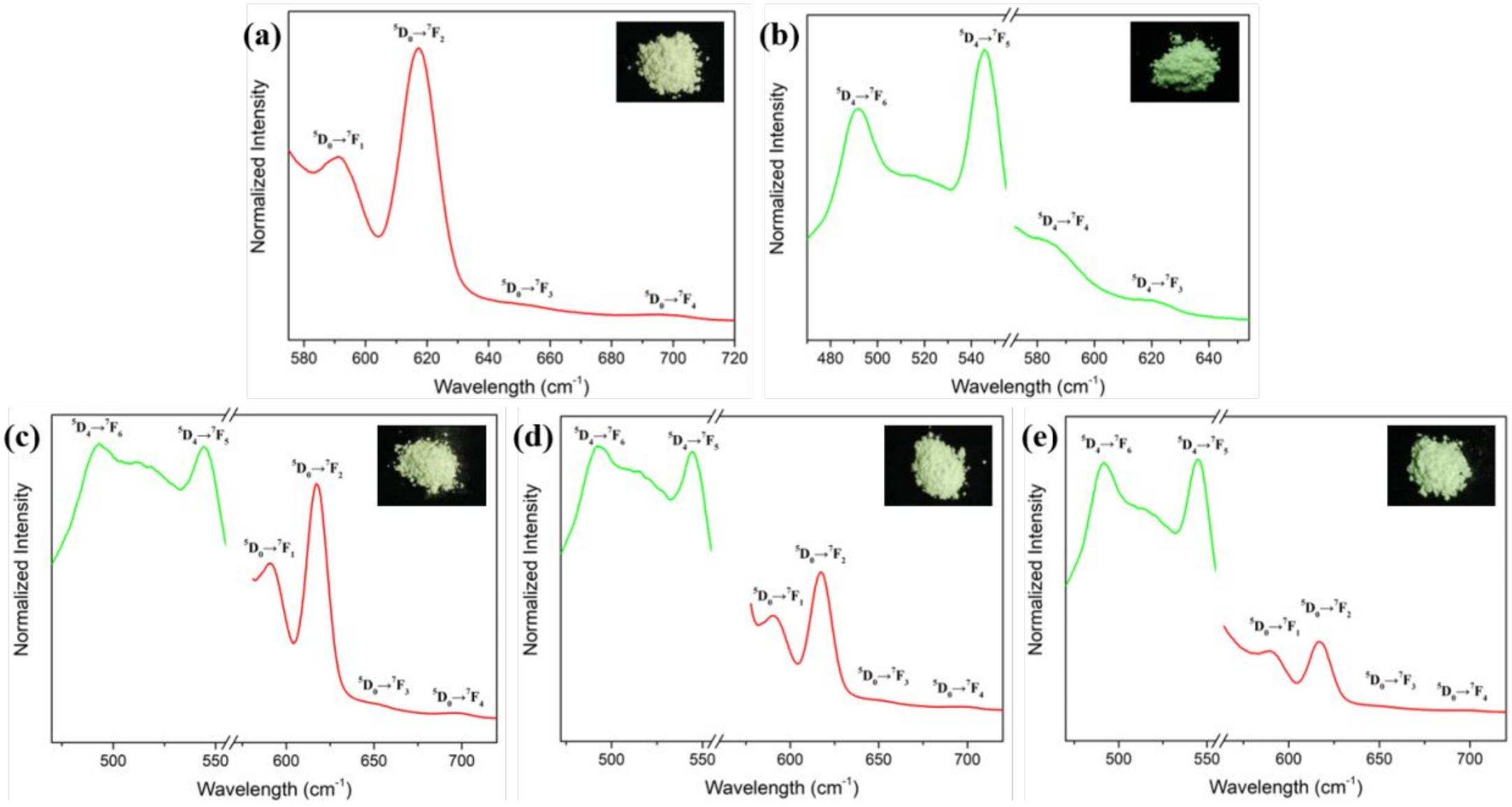

Figure S17. Luminescence properties of compound **NTOU-12c** containing different Eu^{3+}/Tb^{3+} molar ratios: (a) 3/0, (b) 0/3 (c) 2/1, (d) 1/1, and (e) 1/2, under excitation at 270 and/or 300 nm.

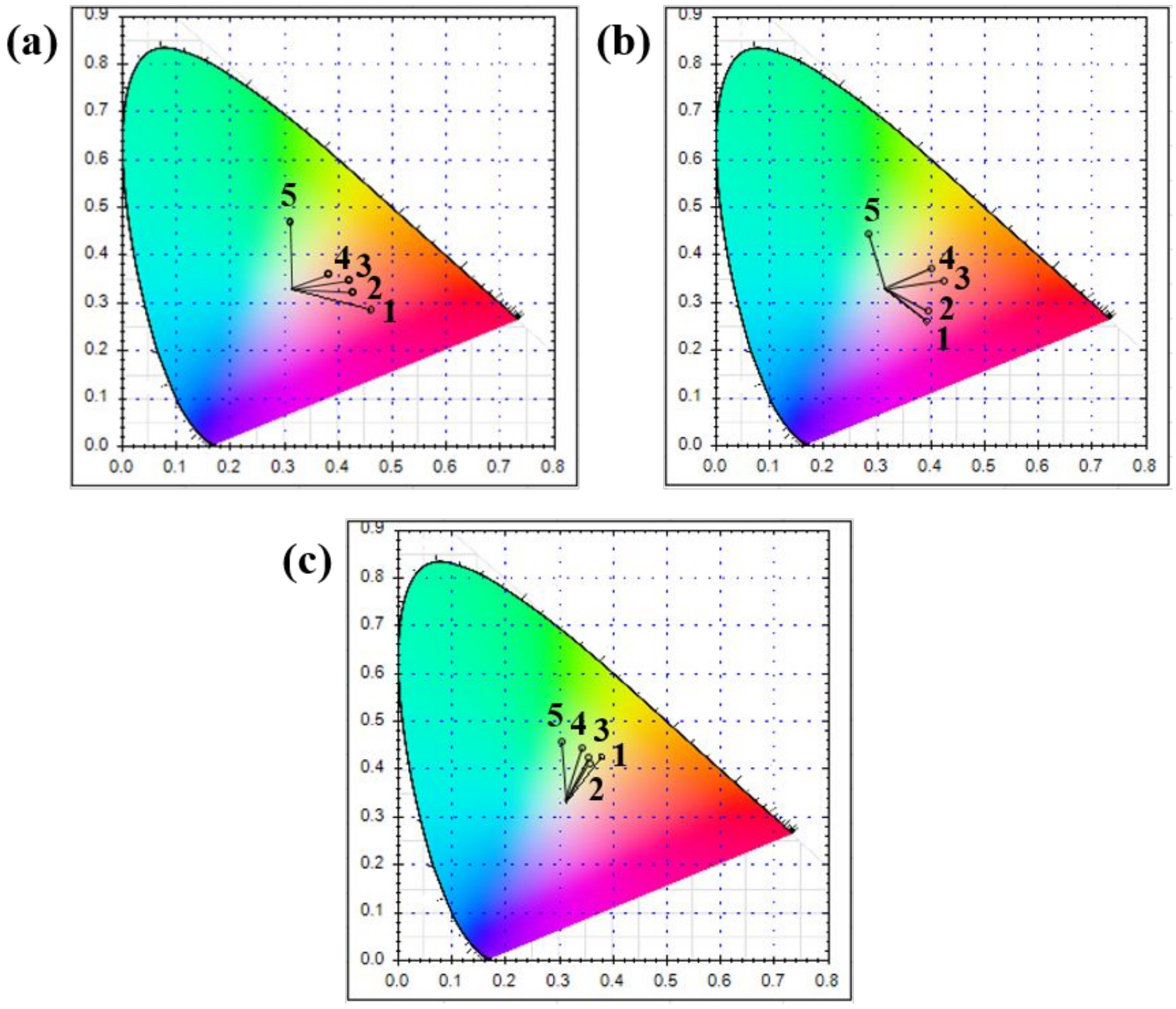

Figure S18. CIE chromaticity diagrams of (a) **NTOU-12a**, (b) **NTOU-12b**, and (c) **NTOU-12c**. The numbered points correspond to different Eu^{3+}/Tb^{3+} molar ratios: (1) 3/0, (2) 2/1, (3) 1/1, (4) 1/2, and (5) 0/3. The measurements were performed under UV irradiation at 254 nm.

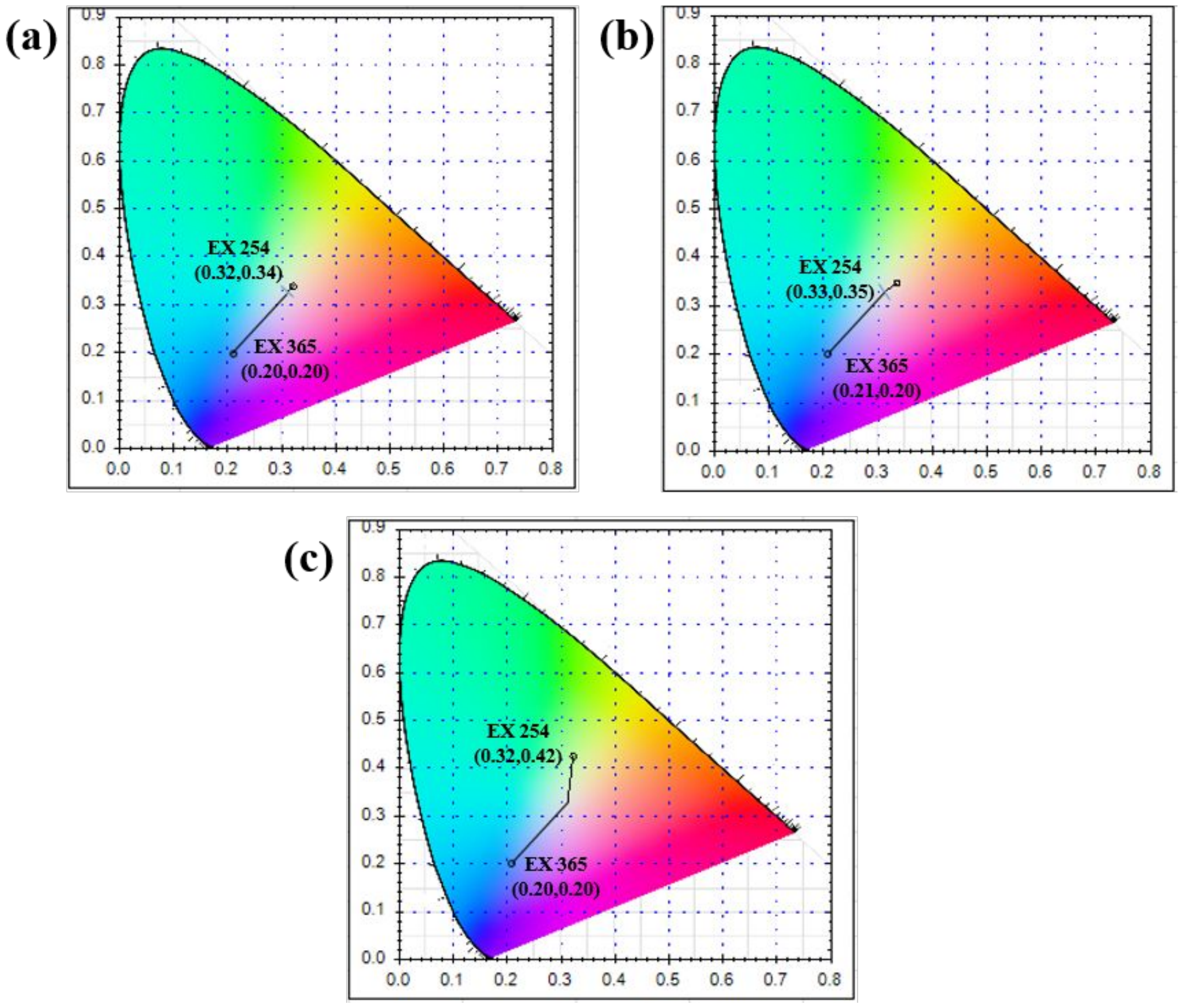

Figure S19. CIE chromaticity diagrams of (a) **NTOU-12a**, (b) **NTOU-12b**, and (c) **NTOU-12c** with an Eu^{3+}/Tb^{3+} molar ratio of 1/4. The measurements were performed under UV irradiation at 254 nm and 365 nm, respectively.

Pristine Sample	Different Eu^{3+}:Tb^{3+} Molar Ratios				
	3:0	2:1	1:1	1:2	0:3
NTOU-12a	(0.46,0.29)	(0.42,0.32)	(0.41,0.35)	(0.38,0.35)	(0.32,0.47)
NTOU-12b	(0.39,0.26)	(0.38,0.29)	(0.39,0.32)	(0.39,0.37)	(0.28,0.45)
NTOU-12c	(0.38,0.43)	(0.35,0.41)	(0.35,0.42)	(0.35,0.43)	(0.30,0.45)

Table S1. CIE coordinates of the title compounds with varying Eu^{3+}/Tb^{3+} molar ratios under excitation at 254 nm.

Pristine Sample	NTOU-12a	NTOU-12b	NTOU-12c
Eu^{3+}	4.76 mg/g	5.93 mg/g	3.47 mg/g
Tb^{3+}	10.12 mg/g	11.82 mg/g	6.93 mg/g

Table S2. ICP-OES analysis of Eu and Tb content for the title compounds after adsorption experiments containing a Eu^{3+}/Tb^{3+} molar ratio of 1/4.
